# Combined Dihydroartemisinin and Eupatilin Suppress Prostate Cancer through AR-Associated Ferroptosis and Modulation of Macrophage–Tumor Crosstalk

**DOI:** 10.34133/research.1331

**Published:** 2026-06-23

**Authors:** Linfeng Wang, Hao He, Congfeng Lei, Siyuan Liu, Yang Cao, Lei Yang, Jiang Yu, Ziling Wei, Xiang Li, Qiuchen Li, Rui Sun, Gaojie Zhang, Yong Huang, Wenjun Chen, Gongjia Qiu, Yingying Gao, Weiyang He, Bowen Xie, Chunxia Ma, Ziyang Zeng, Jiayu Liu

**Affiliations:** ^1^ Department of Urology, The First Affiliated Hospital of Chongqing Medical University, Chongqing, China.; ^2^ School of Public Health, Chongqing Medical University, Chongqing, China.; ^3^ School of Clinical Medicine, Chongqing Medical University, Chongqing, China.; ^4^ Department of Clinical Laboratory, Banan Hospital of Chongqing Medical University, Chongqing, China.; ^5^ School of Pediatrics, Chongqing Medical University, Chongqing, China.; ^6^ Institute of the History of Chinese Medicine and Medical Literature, China Academy of Chinese Medical Sciences, Beijing, China.; ^7^ Shandong Analysis and Test Center, Qilu University of Technology (Shandong Academy of Sciences), Jinan, China.; ^8^ The First Affiliated Hospital of Chongqing University of Chinese Medicine, Chongqing Traditional Chinese Medicine Hospital, Chongqing, China.

## Abstract

Persistent androgen receptor (AR) signaling, metabolic alterations, and changes in the immune microenvironment are contributing factors to the progression of prostate cancer (PCa); however, therapeutic strategies capable of simultaneously targeting these interconnected vulnerabilities remain limited. Here, we identify a combinatorial regimen consisting of dihydroartemisinin and eupatilin (D&E) as a suppressor of PCa progression through coordinated disruption of tumor-intrinsic survival signaling and protumor immune interactions. Functionally, D&E suppressed the proliferation of PCa cells. Mechanistically, D&E induces ferroptosis in PCa cells, as evidenced by lipid peroxidation, intracellular iron accumulation, malondialdehyde elevation, and glutathione reduction. We further identified AR as an important target of D&E and found that it promotes resistance to ferroptosis by sustaining the SLC7A11 antioxidant axis through transcriptional activation of SLC7A11. Upstream, D&E inhibited the NF-κB subunit p65, which transcriptionally maintains AR expression, thus establishing an NF-κB/AR/SLC7A11 signaling cascade underlying ferroptosis resistance in PCa. Beyond tumor-intrinsic effects, D&E also remodels the tumor microenvironment. Specifically, AR-high tumor cells were found to facilitate M2-like macrophage polarization. In turn, M2-like macrophages could secrete SPP1, which potentially augments malignant properties and AR signaling in tumor cells via the CD44 pathway, thereby constituting a protumorigenic positive feedback loop. Notably, D&E attenuated the M2-like phenotype, reduced SPP1 secretion, and reduced macrophage-mediated promotion of PCa proliferation. In immunocompetent mouse models, D&E further synergized with anti-PD-L1 to suppress PCa progression. Together, these findings indicate that D&E exert antitumor effects in PCa by promoting ferroptosis-associated cell death and modulating macrophage-associated microenvironmental signaling, with potential therapeutic relevance particularly in AR-positive PCa.

## Introduction

Prostate cancer (PCa) represents a formidable challenge in global public health and serves as a classic paradigm for acquired therapeutic resistance in solid tumors [1]. Although sequential interventions involving androgen deprivation therapy (ADT) and inhibitors targeting the androgen receptor (AR) signaling axis have, to some extent, prolonged the survival rate of patients with metastatic castration-sensitive prostate cancer (CSPC) [2], the inexorable evolutionary trajectory toward lethal castration-resistance prostate cancer (CRPC) remains unbroken [3,4]. The inevitable emergence of therapeutic resistance, the suboptimal treatment responses observed in patients with advanced disease, and treatment-related toxicities collectively underscore the urgent clinical need for alternative therapeutic agents.

As a major reservoir of bioactive molecules, traditional Chinese medicine has gained broad recognition for its antitumor potential [5]. *Artemisia annua* L. and *Artemisia argyi* H. Lév. & Vaniot are 2 representative members of the Asteraceae family with well-established medicinal value [6,7]. Artemisinin is a sesquiterpene lactone compound purified from *A. annua* and is best known for its potent antimalarial activity [6]. Dihydroartemisinin (DHA), a derivative of artemisinin, exhibits improved solubility and greater antitumor activity, and its mechanisms of action are distinctly pleiotropic [8]. Notably, nanoscale DHA delivery systems have been shown to profoundly remodel the tumor immune microenvironment, driving tumor-associated antigen exposure and orchestrating robust T-cell immunity [9]. Eupatilin, a pharmacologically active flavonoid extracted from *Artemisia* species, has emerged as a versatile suppressor of oncogenic networks. Evidence from previous studies indicates that the antitumor activity of eupatilin in different cancers is mediated by multiple mechanisms. In hepatocellular carcinoma, eupatilin markedly inhibits angiogenesis and metastasis by down-regulating the matrix metalloproteinase-2 (MMP-2) and vascular endothelial growth factor (VEGF) signaling pathways [10]. In gastric cancer cells, it further suppresses tumor angiogenesis and limits growth through the blockade of STAT3-mediated VEGF expression [11]. Eupatilin limits the migratory behavior of PCa cells by affecting phosphatase and tensin homolog (PTEN) and nuclear factor kappa B (NF-κB) signaling cascades [12].

Accumulating evidence indicates that both DHA and eupatilin exhibit considerable potential to induce ferroptosis in cells [13,14]. Ferroptosis has recently been identified as a distinct form of regulated cell death and has emerged as one of the important mechanisms by which natural products from traditional Chinese medicine exert antitumor effects [15,16]. Mechanistically, DHA actively triggers ferroptosis by accelerating the lysosomal degradation of ferritin, thereby dramatically expanding the intracellular labile iron pool [17,18]. Eupatilin may likewise possess the capacity to induce ferroptosis in tumor cells. Several studies have shown that flavonoid compounds can trigger ferroptosis in PCa cells [19–21], and eupatilin has been reported to suppress ferroptosis and alleviate secondary brain injury by activating SOX2-dependent SLC7A11 expression, suggesting that it may participate in ferroptosis-related pathways through regulation of SLC7A11 [14].

Despite these encouraging findings, studies of DHA and eupatilin in PCa remain relatively limited. The present work revealed that combined DHA and eupatilin treatment promotes AR-dependent ferroptosis and provides a theoretical basis for herbal combination strategies targeting this pathway in PCa treatment.

## Results

### DHA combined with eupatilin induces ferroptosis in PCa cells

**Fig. 1.**
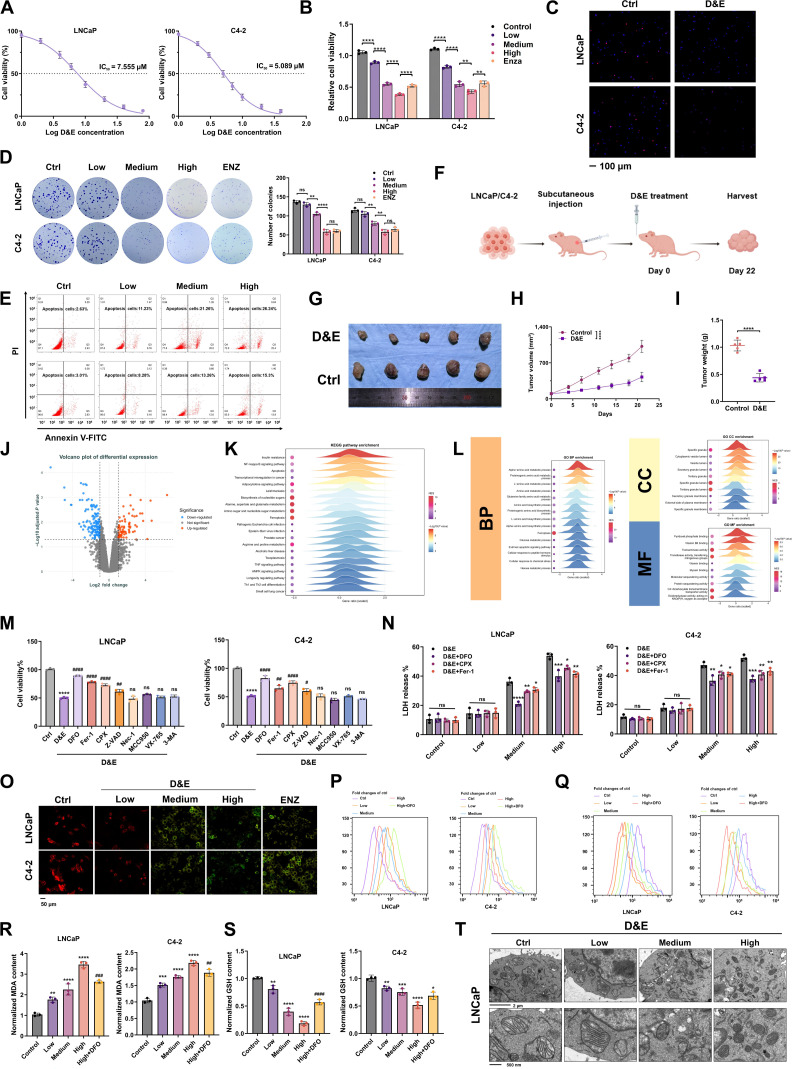
Combined treatment with dihydroartemisinin (DHA) and eupatilin induces ferroptosis. (A and B) After CCK-8 assays, IC_50_ curves (A) were generated to evaluate cell viability (B) in PCa cells following combined treatment with variant concentrations of DHA and eupatilin (D&E). (C) Ki-67 immunofluorescence showed changes in the proliferative status of PCa cell lines after treatment with a high dose of D&E. (D and E) Colony formation assay and flow cytometry were used to analyze cell proliferation and apoptosis in PCa cells treated with different concentrations of D&E and 10 μM enzalutamide. (F to I) Subcutaneous xenografts were generated in male nude BALB/c mice (*n* = 5 per group). After random grouping, mice in the D&E group received combination therapy: DHA (25 mg/kg, 200 μl) was administered intraperitoneally daily, and eupatilin (10 mg/kg, 80 μl) was administered intraperitoneally every 2 days for 3 consecutive weeks (F). Mice were sacrificed on day 22 after treatment, and tumors were harvested (G). Tumor volume was monitored continuously during treatment (H), and final tumor weight was calculated (I). (J to L) RNA sequencing was performed in LNCaP cells after treatment with a high dose of D&E for 24 h in the D&E and Mock groups (*n* = 3 per group). Volcano plots were constructed based on *P* value (*P* < 0.05) and fold change (J). KEGG (K) and GO analyses (L) were conducted on 233 significantly up-regulated differentially expressed genes after D&E treatment. (M and N) LNCaP and C4-2 cells were treated for 48 h with a high dose of D&E plus the indicated cell death inhibitors, followed by CCK-8 measurement of cell viability. (M) LDH levels in these cells were measured using an LDH detection kit (N). (O to T) LNCaP and C4-2 cells were exposed to a high dose of D&E for 48 h in the presence or absence of DFO (100 μM). Lipid ROS levels were detected using the C11-BODIPY-581/591 probe and are shown as representative fluorescence images (O) and flow cytometry results (P). Intracellular iron levels were assessed using the PGSK probe in combination with flow cytometry (Q). Cell lysates were simultaneously collected, and the levels of MDA (R) and GSH (S) were measured using commercial assay kits. (T) Representative TEM images of LNCaP cells treated with vehicle control or increasing concentrations of D&E (low, medium, and high dose). Scale bars, 2 μm (top panels) and 500 nm (bottom panels). Data are presented as the mean ± SD. *P < 0.05, **P < 0.01, ***P < 0.001, ****P < 0.0001, and n.s. indicate no significant difference compared with the control group; #*P* < 0.05, ##*P* < 0.01, ###*P* < 0.001, and ####*P* < 0.0001 indicate significant differences compared with the high dose group or the D&E group. All experiments were repeated 3 times independently.

Dose-dependent inhibition of PCa cell growth by both DHA and eupatilin was observed in vitro (Fig. S1A and B). Notably, when DHA and eupatilin were administered in combination (D&E), the antiproliferative effect was enhanced (Fig. 1A). Bliss analysis showed synergistic effects in both cell lines, with positive Bliss excess at most doses and peak synergy at 10 μM in LNCaP (27.96 pp) and 7 μM in C4-2 (30.58 pp). Consistently, Chou–Talalay analysis showed CI < 1 at all tested doses in LNCaP and at most doses in C4-2, with the lowest CI values at Fa = 0.628 (CI = 0.475) and Fa = 0.775 (CI = 0.436), respectively (Fig. S1C to E). As a second-generation nonsteroidal AR antagonist and one of the principal targeted treatments for PCa, enzalutamide was employed as a positive reference to evaluate the efficacy of D&E. Results from Ki-67 staining and cell viability assays showed that high-dose D&E inhibited PCa cell proliferation at a level comparable to enzalutamide, suggesting its possible therapeutic relevance in PCa management (Fig. 1B and C). In addition, to further examine how combined D&E treatment influences the malignant characteristics of PCa cells, colony formation assays and flow cytometry were conducted, demonstrating a dose-dependent reduction in proliferation accompanied by an increase in apoptosis (Fig. 1D and E). These findings indicate that D&E suppresses the malignant behavior of PCa cells in vitro. Consistently, in tumor-bearing mice, D&E treatment reduced both the weight and volume of xenografted tumors (Fig. 1F to I). Notably, high-dose D&E exhibited minimal cytotoxicity toward normal tissues, as evidenced by unchanged mice body weight (Fig. S1F) and preserved morphology of major organs (Fig. S1G).

We performed transcriptomic RNA sequencing (RNA-seq) analysis in PCa cell lines following D&E treatment. Differential expression analysis identified 233 up-regulated genes in D&E-treated cells, and subsequent Kyoto Encyclopedia of Genes and Genomes (KEGG) and Gene Ontology (GO) analyses revealed that these genes were predominantly related to ferroptosis-related pathways (Fig. 1J to L). Preclinical evidence has increasingly pointed to ferroptosis as one of the important mechanisms by which certain bioactive compounds derived from traditional Chinese medicine mediate tumor suppression [13,22,23]. We therefore hypothesized that ferroptosis likewise serves as a key mechanism underlying the inhibitory effect of D&E on PCa.

We next explored the pattern of tumor cell death triggered by D&E, with a particular focus on the contribution of ferroptosis. To this end, cells were coincubated with D&E in the presence of a panel of inhibitors targeting distinct cell death pathways, and cytotoxicity was reevaluated by Cell Counting Kit-8 (CCK-8) assay. Deferoxamine (DFO), ferrostatin-1 (Fer-1), and ciclopirox (CPX) were used to inhibit ferroptosis; they markedly reversed D&E-induced cytotoxicity in PCa cells, whereas inhibitors of other cell death pathways showed only limited effects. The apoptosis inhibitor z-VAD-FMK also produced a modest protective effect (Fig. 1M). Consistently, D&E-induced lactate dehydrogenase (LDH) release was also attenuated by DFO, CPX, and Fer-1 (Fig. 1N).

As anticipated, D&E treatment elicited several hallmark features of ferroptosis, including increased lipid peroxidation (Fig. 1O and P), iron accumulation (Fig. 1Q), malondialdehyde (MDA) elevation (Fig. 1R), and reduction of glutathione (GSH) (Fig. 1S), all of which were rescued as the dose increased. Transmission electron microscopy (TEM) further revealed pronounced mitochondrial abnormalities following D&E treatment (Fig 1R and Fig. S1H). Taken together, these results support the notion that D&E effectively induces ferroptosis in PCa cells.

### AR is a potential target of D&E in the treatment of PCa

**Fig. 2.**
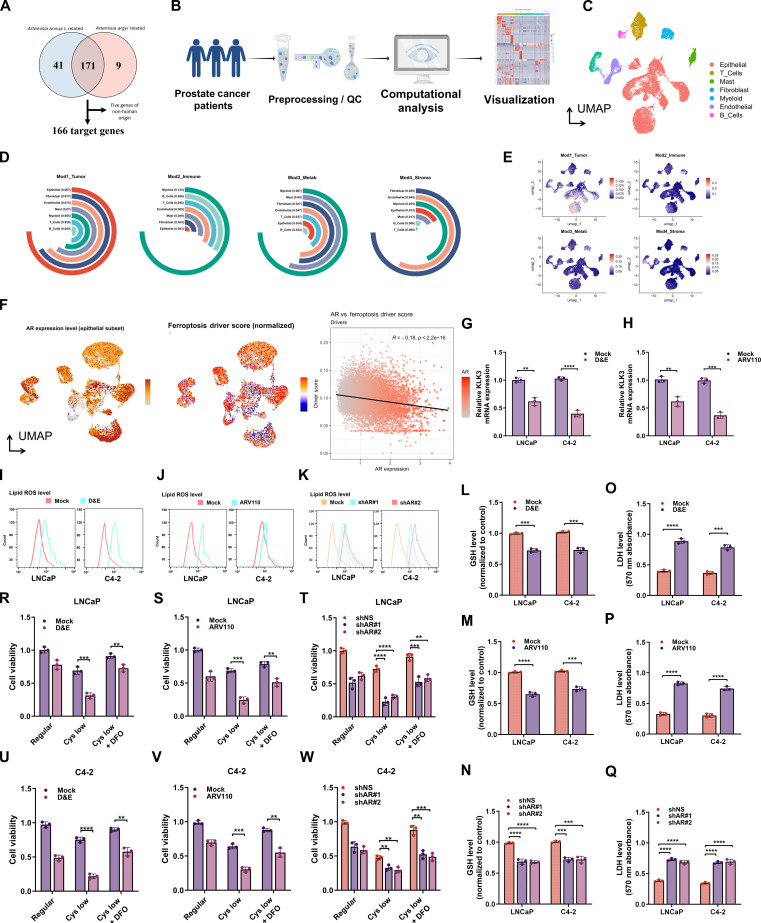
Prediction and validation of AR as a therapeutic target of D&E. (A) Venn diagram showing the 166 potential common targets of *Artemisia argyi* (Aiye) and *Artemisia annua* (Qinghao) retrieved from https://tcmsp-e.com/. (B) Analysis workflow for the single-cell dataset GSE141445. (C) UMAP visualization of single-cell data after PCA dimensionality reduction, based on marker genes of distinct cell types. (D and E) The 166 potential targets were functionally divided into 4 modules. Scoring was performed at the single-cell level using the AUCell (v1.30.1) algorithm. Scores of the 4 modules in different cell clusters (D) and their UMAP distribution (E) are displayed. (F) The “Epithelial” cell cluster was extracted from single-cell data, and AR expression in this cluster was labeled. Ferroptosis Driver score distribution in the Epithelial cluster was visualized by UMAP using the AUCell (v1.30.1) algorithm; a redder color indicates a higher Ferroptosis Driver score. The correlation between AR level and Ferroptosis driver score in the epithelial cell cluster was examined using Pearson correlation test. (G and H) LNCaP and C4-2 cells were incubated with vehicle, high-dose D&E (G), or ARV110 (H) for 48 h, and KLK3 mRNA levels were then examined by qRT-PCR. (I to Q) LNCaP and C4-2 cells were treated with vehicle, high-dose D&E, or ARV110, or alternatively transduced with nonspecific shRNA (shNS) or AR-targeting shRNAs. After 48 h, lipid peroxidation was evaluated by C11-BODIPY staining followed by flow cytometry (I to K), whereas intracellular GSH (L to N) and LDH (O to Q) levels were determined using the corresponding assays. (R to W) LNCaP (R to T) and C4-2 (U to W) cells were maintained in regular or cystine-restricted medium (2 μmol/l), with or without DFO, and subjected to vehicle, ARV110, D&E, lentiviral AR shRNAs, or control shRNA for 72 h, after which cell viability was assessed. Data are presented as mean ± SD. *P < 0.05, **P < 0.01, ***P < 0.001, ****P < 0.0001; n.s., not significant versus the control group. Three independent biological experiments were performed.

To further identify the specific targets through which D&E regulates the malignant behavior of PCa, we retrieved 166 putative human targets shared by *A. annua* L. and *A. argyi* (Fig. 2A) from the Traditional Chinese Medicine Systems Pharmacology (TCMSP) database (https://www.tcmsp-e.com/index.php) by querying the keywords “*aiye*” and “*qinghao*”. These genes were highly enriched in tumor-related pathways, including those associated with PCa (Fig. S2A). We next categorized these genes on the basis of their putative biological functions and defined 4 functional modules: M1, PCa tumor cell-intrinsic programmers (AR/proliferation/survival signaling); M2, immune and inflammatory processes (macrophages/immune cells; cytokines/chemokines/signaling); M3, lipid peroxidation, oxidative stress, and detoxification metabolism (NRF3–ARE/lipoxygenase pathways/drug metabolism); and M4, stroma–vasculature–coagulation and extracellular matrix remodeling (endothelial adhesion/fibrinolysis/collagens/MMPs) (Table S3). These findings suggest that *A. annua* L. and *A. argyi* may have the potential to affect multiple cell groups of the tumor microenvironment (TME).

Single-cell transcriptomic analysis has become an indispensable step in exploring gene mechanisms in PCa research [24]. To delineate the more refined intercellular regulatory landscape underlying the effects of D&E in PCa, we incorporated the pathway-clustered target genes into single-cell analyses. We obtained and analyzed a single-cell dataset of 13 patients with PCa from the Gene Expression Omnibus (GEO) database (Fig. 2B and C). The distribution of genes across the 4 functional modules was then visualized at single-cell resolution (Fig. 2D and E).

We further interrogated the transcriptomic profiles of PCa cells following D&E treatment, with particular attention to genes that were down-regulated in response to treatment. Among the candidate targets identified above, AR emerged as the most markedly reduced gene at the mRNA level after D&E exposure, raising the possibility that it contributes to D&E-induced ferroptosis in PCa cells (Fig. S2B). Consistent with this notion, AR expression was decreased at both the transcriptional and protein levels in D&E-treated cells (Fig. S2C and D), suggesting that D&E suppresses AR signaling in PCa cell lines. Furthermore, single-cell transcriptomic data showed predominant AR expression in PCa tumor cells, with a negative correlation to the ferroptosis score (Fig. 2F and Fig. S2E). We next sought to determine how AR suppression relates to lipid peroxidation and ferroptosis in PCa cells under different experimental conditions. As expected, AR inhibition down-regulated KLK3, a canonical AR target gene (Fig. 2G and H and Fig. S2F and G). In addition, treatment with ARV-110 together with 2 independent AR-specific shRNAs showed that AR suppression enhanced D&E-induced lipid peroxidation in PCa cells through both pharmacological inhibition and genetic silencing (Fig. 2I to K). AR inhibition also decreased GSH levels while increasing soluble LDH, indicating compromised cellular integrity (Fig. 2L to Q). We reduced the cystine supply in the culture medium to determine whether D&E-induced AR-dependent cell death is associated with the core metabolic network of ferroptosis. Reducing cystine availability impaired the viability of cells, and this effect was markedly enhanced by D&E treatment, ARV-110, or AR shRNA. By contrast, DFO rescued the loss of viability induced by cystine deprivation (Fig. 2R to T). Collectively, these findings suggest that AR is likely a key target of D&E in PCa and that D&E induces ferroptosis in PCa cells, at least partly through an AR-dependent mechanism.

### AR binds to and transcriptionally activates SLC7A11

**Fig. 3.**
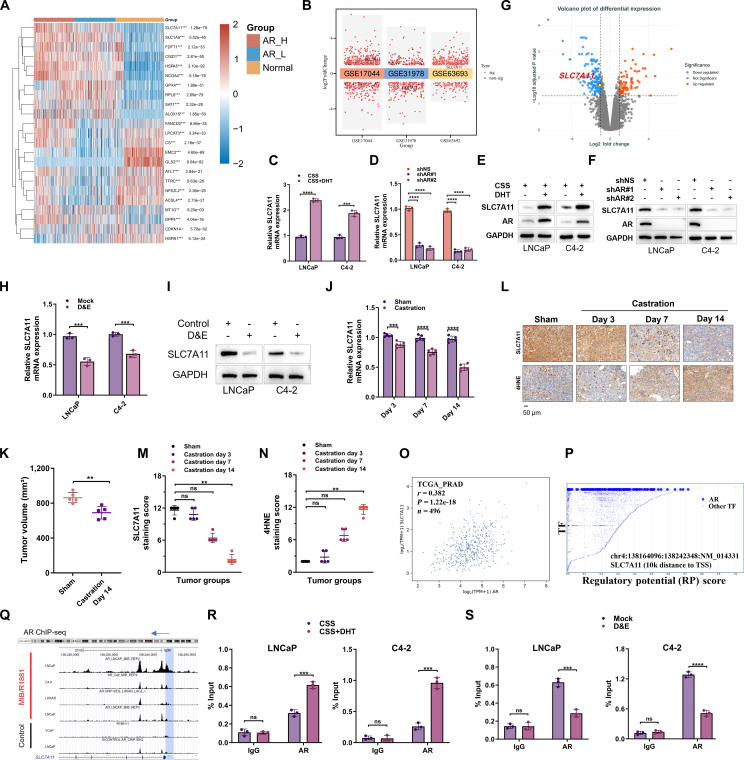
AR binds to and induces SLC7A11 expression. (A) Heatmap showing the expression distribution of AR and ferroptosis-related genes. Transcriptome data of PCa were obtained from the TCGA database. Tumors were divided into AR_H and AR_L groups according to the median expression of AR. Comparisons among the 3 groups were performed using the Kruskal–Wallis test. (B) Multiple volcano plots showing the differential analysis results of GSE17044 (R1881 treatment vs. control), GSE31978 (hormone deprived vs. control), and GSE63692 (R1881 vs. control). The distribution of genes with log_2_foldchange ≤−1 or ≥1 is presented, and the position of SLC7A11 is labeled with black dots. (C and D) qRT-PCR (C) and Western blot (D) analyses showed SLC7A11 expression in LNCaP and C4-2 cells cultured under androgen-deprived conditions and treated with or without DHT (10 nmol/l) for 24 h. (E and F) LNCaP and C4-2 cells were infected for 72 h with lentiviral shNS or AR-specific shRNA, after which SLC7A11 protein and transcript levels were determined by Western blot analysis (E) and qRT-PCR (F). (G) RNA sequencing was performed in LNCaP cells after treatment with a high dose of D&E for 24 h in the D&E and Mock groups (*n* = 3 per group). Volcano plots were constructed based on fold change and *P* value (*P* < 0.05). Yellow dot indicates SLC7A11. (H and I) SLC7A11 expression in LNCaP and C4-2 cells treated in the presence or absence of high-dose D&E for 72 h was examined by qRT-PCR (H) and Western blotting (I). C4-2 xenograft tumors from male mice receiving sham castration or surgical castration (*n* = 5 per group) were analyzed by qRT-PCR. (J) Relative SLC7A11 mRNA expression in tumor tissues from sham-operated and castrated mice at days 3, 7, and 14 after castration, as determined by qRT-PCR. (K to N) Tumor volumes of C4-2 xenografts from mice receiving sham operation or castration for 14 days (K). Representative images (L), together with quantification of SLC7A11 (M) and 4HNE (N) IHC staining, are shown for tumors collected at the indicated time points after castration (*n* = 5 tumors/group). (O) Pearson correlation between AR and SLC7A11 expression in TCGA-PRAD was analyzed using the GEPIA3 database (https://gepia3.bioinfoliu.com/). (P) Potential transcription factors binding to the SLC7A11 genomic region were predicted by integrative analysis of public ChIP datasets using the Cistrome platform. (Q) Public AR ChIP-seq tracks from the Cistrome database revealed AR enrichment at the SLC7A11 locus in PCa cells cultured in CSS medium with or without R1881 or mibolerone. (R and S) AR occupancy at the promoter regions of SLC7A11 was further examined by qChIP-PCR with or without DHT (R) or high-dose D&E (S). All data are shown as mean ± SD, *P < 0.05, **P < 0.01, ***P < 0.001, ****P < 0.0001, n.s., not significant vs. control group. Three independent biological experiments were performed.

To elucidate how the AR regulates ferroptosis in PCa cells, we first analyzed the relationship between AR and canonical ferroptosis-related molecules using samples from The Cancer Genome Atlas (TCGA)-prostate adenocarcinoma (PRAD) and Genotype-Tissue Expression (GTEx) databases (AR_H = 249, AR_L = 249, NC = 279) (Fig. 3A). We then examined RNA-seq datasets from PCa models in which AR was activated by R1881 (GSE17044 and GSE63693). These analyses indicated that activation of the androgen–AR axis was associated with alterations in ferroptosis-related and lipid metabolism (Fig. S3A). Notably, SLC7A11 was consistently up-regulated in parallel with AR activation. Moreover, we observed reduced SLC7A11 expression in RNA-seq datasets from androgen-deprived PCa cells and in single-cell datasets modeling AR depletion, suggesting that SLC7A11, a known suppressor of ferroptosis, may be regulated by AR in PCa (Fig. 3B and Table S4). Based on prior knowledge, we investigated the relationship between AR and SLC7A11 under D&E treatment. We observed a potential regulatory relationship between AR and SLC7A11 (Fig. 3C to F). Consistent with these findings, RNA-seq data showed that D&E treatment down-regulated SLC7A11 mRNA expression in PCa cells (Fig. 3G). To extend this analysis at the molecular level of ferroptosis, we also examined GPX4. Whereas D&E treatment reduced both the transcription and protein levels of SLC7A11, GPX4 appeared to be regulated by D&E primarily at the protein level (Fig. 3H and I and Fig. S3B to E). In vivo experiments further showed that the expression of AR and SLC7A11 was markedly down-regulated in tumor tissues from castrated CDX mice (Fig. 3J to N). Taken together, these findings suggest that, in the AR-suppressed state established by D&E, SLC7A11 may also be coordinately inhibited.

Given its established role as a transcriptional activator, together with the changes observed at the mRNA level, we reasoned that AR might promote the transcription of SLC7A11. Using TF Target Finder (https://jingle.shinyapps.io/TF_Target_Finder/), predictions across the CHEA, GTRD, ChIP-Atlas, hTFtarget and KnockTF databases consistently identified AR as a putative transcription factor for SLC7A11 in PCa and indicated a positive correlation between AR and SLC7A11 expression (cor_r > 0.3) (Fig. 3O, Fig. S3F, and Table S5). In agreement with the expression data, chromatin immunoprecipitation sequencing (ChIP-seq) for candidate transcription factors identified AR as one of the factors with strong transactivation potential at the SLC7A11 locus (Fig. 3P), supporting the possibility that activated AR regulates SLC7A11 expression through direct occupancy of this locus. To examine this further, we extracted and analyzed AR ChIP-seq datasets, which showed that AR activation increased AR occupancy across the transcriptionally active region of the SLC7A11 locus in PCa cell lines (Fig. 3Q). We further verified by chromatin immunoprecipitation (ChIP)-qPCR that androgen stimulation increased AR binding at the SLC7A11 promoter region (Fig. 3R). In contrast, D&E treatment reduced AR enrichment at the SLC7A11 promoter region (Fig. 3S). Our results imply that AR may facilitate SLC7A11 expression through direct binding to its genomic locus, and that this occupancy can be disrupted by D&E treatment or androgen deprivation.

### D&E promotes ferroptosis in PCa by suppressing the AR/SLC7A11

**Fig. 4.**
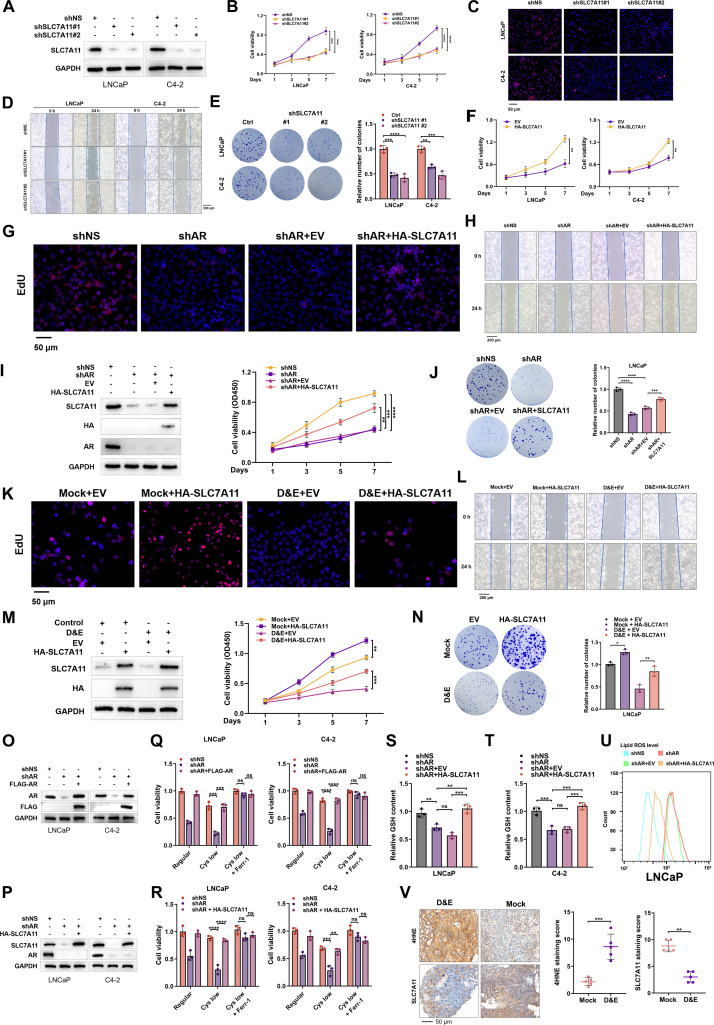
D&E suppresses AR/SLC7A11 signaling and promotes ferroptosis in PCa cells. (A and B) Stable LNCaP and C4-2 cells expressing shSLC7A11 were subjected to Western blotting (A) and CCK-8 assays (B). (C) Representative EdU staining images showing the effect of SLC7A11 knockdown on cell proliferation in LNCaP and C4-2 cells. (D) Representative wound-healing assay images showing the migratory ability of LNCaP and C4-2 cells after SLC7A11 knockdown at 0 and 24 h. Scale bar, 200 μm. (E) Colony formation capacity of cells expressing shSLC7A11 (vs shNS). (F) Viability of the indicated cell lines after HA-SLC7A11 transfection was determined at different time points. (G to J) LNCaP cells were transduced for 72 h with lentiviruses carrying the indicated shRNAs or expression constructs, followed by EdU (G), wound-healing assay (H), Western blotting and CCK-8 assays (I), and colony formation assays with quantitative analysis (J). (K to N) LNCaP cells expressing the indicated constructs were cultured in the presence or absence of D&E and then subjected to EdU (K), wound-healing assay (L), Western blotting and CCK-8 analysis (M), and colony formation assays (N). (O and P) The indicated proteins were examined by Western blotting in LNCaP and C4-2 cells 72 h after transfection with the specified plasmids and/or shRNAs. (Q and R) Viability of transfected LNCaP and C4-2 cells was determined after culture in low-cystine medium (2 μM). (S to U) Cells were cultured in cystine-restricted medium (2 μmol/l) for 48 h, followed by assessment of cell viability (S), intracellular GSH levels (T), and lipid peroxidation by C11-BODIPY staining with flow cytometric detection (U). (V) 4HNE and SLC7A11 IHC staining in xenograft tumors from mice receiving D&E or control treatment for 14 days. All data are shown as mean ± SD, *P < 0.05, **P < 0.01, ***P < 0.001, ****P < 0.0001, n.s., not significant. One-way ANOVA followed by Tukey’s post hoc test evaluated significance among multiple groups. Three independent biological experiments were performed.

SLC7A11 transports extracellular cystine into the cell to support GSH production, whereas GPX4 relies on GSH to eliminate lipid peroxides, thereby preventing ferroptotic cell death [25]. After modulating SLC7A11 mRNA levels, we found that its expression influenced the growth of PCa cells (Fig. 4A to F). In addition, restoration of SLC7A11 expression rescued the decrease in cell viability caused by AR silencing (Fig. 4G to J) and reversed the inhibitory effects of D&E on cell viability and proliferation (Fig. 4K to N). When cystine supply was reduced, reexpression of either AR or SLC7A11 was able to rescue the cell suppression mediated by D&E (Fig. 4O to R). We further observed that overexpression of SLC7A11 alleviated the D&E-induced reduction in GSH and increase in lipid peroxidation (Fig. 4S to U). In an LNCaP cell-derived CDX mouse model, immunohistochemical staining for 4-hydroxynonenal further showed increased lipid peroxidation in tumor tissues after D&E treatment, while SLC7A11 expression was concomitantly decreased (Fig. 4V). Together, these data suggest that ferroptosis may at least partially contribute to the cell death induced by D&E both in vitro and in vivo.

### D&E inhibits AR through an NF-κB-dependent mechanism

To further clarify the pathway through which D&E suppresses AR, we examined upstream regulatory mechanisms that might account for this effect. Transcriptomic analyses indicated that the NF-κB pathway was inhibited following D&E treatment (Fig. 1J to L). In parallel, we had observed that D&E reduced AR expression at both the transcriptional and protein levels, raising the possibility that these changes might result from altered activity of upstream transcriptional regulators (Fig. S2C and D). Notably, NF-κB signaling is known to be constitutively activated in androgen-independent PCa cells and to promote AR accumulation in PCa [26]. On this basis, we hypothesized that D&E-mediated inhibition of NF-κB might contribute to the reduction in AR expression. We first explore the regulatory effects of NF-κB-related factors on AR, and we searched for putative binding sites within the AR promoter using the JASPAR database (http://jaspar.genereg.net). Sequence analysis showed that P65 (also known as RELA) had the highest predicted binding score among NF-κB-related factors (Fig. S4A). We then examined whether RELA regulates AR transcription and found that overexpression of P65 increased AR expression with D&E treatment (Fig. S4B). In addition, D&E treatment reduced phosphorylated P65 and P65 nuclear localization, and decreased the expression of AR and KLK3, whereas P65 overexpression partially rescued these effects (Fig. S4C and D). Importantly, overexpression of P65 likewise reversed the tumor-suppressive effects of D&E and attenuated D&E-induced lipid peroxidation (Fig. S4E to H). These results suggest that D&E-mediated suppression of AR may depend on P65, at least in part.

Using Cistrome DB, we found that P65 showed regulatory potential toward AR (Fig. S4I). To further support the interaction between P65 and the AR promoter, ChIP assays were performed to examine P65 occupancy at the AR promoter region (Fig. S4J). ChIP analysis suggested that P65 occupied the AR promoter region, and this binding was markedly altered by D&E treatment (Fig. S4J and K). Consistent with these findings, P65 ChIP-seq data [27] (GSE83860) also showed a peak signal within this region of the AR promoter (Fig. S4L). Together, these results suggest that D&E may suppress the interaction between P65 and AR in PCa cells, thereby inhibiting AR expression.

Molecular docking compared the binding of D&E to P65 and AR. The results indicated that D&E has binding potential for both P65 and AR, raising the possibility that NF-κB and AR may function along a shared pathway contributing to ferroptosis resistance. The stronger predicted binding affinity toward P65 may also be consistent with a broader inhibitory effect of D&E on PCa signaling networks (Fig. S4M and N).

### D&E suppresses M2 macrophage polarization and SPP1 secretion in PCa

**Fig. 5.**
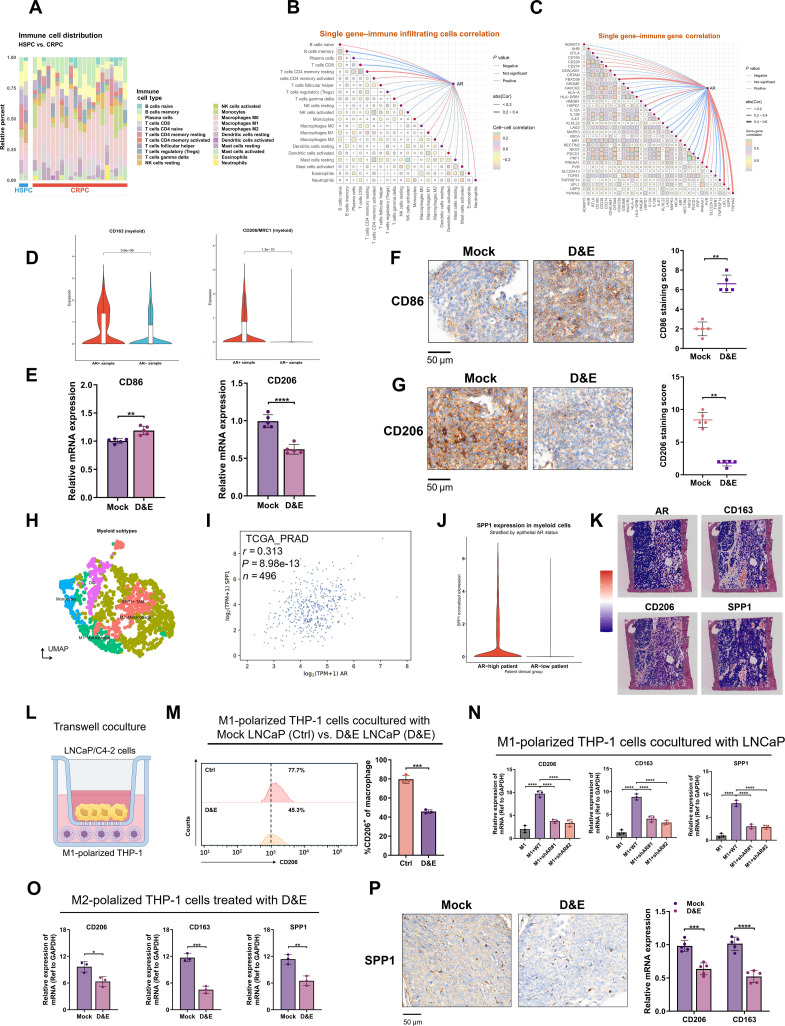
D&E suppresses macrophage M2 polarization and SPP1 secretion in PCa. (A) Immune cell infiltration landscape in HSPC and CRPC samples estimated by deconvolution analysis (GSE32269). Empirical *P* values were calculated using 1,000 permutations, and only samples with *P* < 0.05 were retained. (B and C) Correlation analysis between AR expression and infiltrating immune cell types (B) or immune response-related genes from the MSigDB GOBP_IMMUNE_RESPONSE_TO_TUMOR_CELL gene set (C). Correlations were classified according to significance (*P* < 0.05) and correlation strength. (D) Samples were divided into subgroups based on the median AR expression in the epithelium cell cluster. Violin plots showing CD163 and CD206/MRC1 expression in AR-positive (AR+) and AR-negative (AR−) cell populations at the single-cell level. Expression differences between groups were assessed as indicated by the corresponding *P* values. (E to G) qRT-PCR analysis of the indicated genes in subcutaneous tumors derived from TRAMP-C2 cells in C57BL/6 mice (random assigned, *n* = 5 per group), and tumors were collected 22 days after treatment initiation (E). (F and G) Representative IHC staining and corresponding quantification/IHC scores of CD86 (F) and CD206 (G) in tumor tissues from the indicated groups. Scale bar, 50 μm. (H) UMAP plot of myeloid cell clusters extracted separately from single-cell data shows the distribution of M1-like, M2-like, and SPP1 + TAM cell clusters. (I and J) The correlation between AR and SPP1 expression in TCGA-PRAD was calculated using the GEPIA3 website (I). Violin plots illustrating differential SPP1 expression in myeloid cell clusters between the AR-high and AR-low groups at single-cell resolution (J). (K) Spatial transcriptomic maps (GSE230282) showing the distribution of AR, CD163, CD206, and SPP1 expression in PCa tissue. Feature plots indicate the spatial localization and relative expression levels of the indicated genes across the tissue section. Red indicates high expression, and blue indicates low expression. (L) Diagram of the Transwell coculture model established with ex vivo induced M1-polarized THP-1 cells and LNCaP or C4-2 cells. (M) CD206 expression in M1-polarized THP-1 cells cocultured with LNCaP cells treated with or without a high dose of D&E, as determined by flow cytometry. (N) qRT-PCR analysis of the M2-associated TAM markers CD206 and CD163, together with SPP1, in M1-polarized THP-1 cells cocultured with WT or shAR (#1 and #2) LNCaP cells. (O) Relative mRNA expression of CD206, CD163, and SPP1 in vivo programmed M2-like THP-1 cells treated with high dose D&E. Data are shown as mean ± SD. (P) IHC and qRT-PCR analysis of the indicated genes in subcutaneous tumors derived from TRAMP-C2 cells in C57BL/6 mice with or without D&E treatment. All data are shown as mean ± SD, *P < 0.05, **P < 0.01, ***P < 0.001, ****P < 0.0001, n.s., not significant vs. control group, Student *t* test. Multiple groups: one-way ANOVA + Tukey’s post hoc test. Three independent biological experiments were performed.

PCa is commonly defined as an immunologically “cold” tumor, owing to its relatively low tumor mutational burden, limited antigenicity, an immunosuppressive microenvironment, and the presence of multiple immune evasion mechanisms [28,29]. Single-cell analyses showed that module 2 genes, which were enriched for immune-related targets, were expressed predominantly by myeloid cells (Fig. 2D), prompting us to further investigate the effects of D&E on the immune microenvironment of PCa from the perspective of myeloid populations. Current evidence generally suggests that progression of PCa following AR pathway inhibition is accompanied by remodeling of myeloid cells, particularly macrophages [30,31]. Consistent with this, immune infiltration analysis of a public PCa dataset (GSE32269) indicated that macrophage infiltration was greater in CRPC than in hormone-sensitive PCa (Fig. 5A). Analysis using the CIBERSORT algorithm likewise supported a positive correlation between AR expression and macrophage infiltration in PCa. Spatial transcriptomic data further showed that the M2-like markers CD206 and CD163 were expressed at higher levels than the M1-like marker CD86 in PCa tissues (Fig. 5B and Fig. S5A and B). AR expression was also substantially associated with several immune-related genes, including PDCD1, CD274, HAVCR2, and LAG3 (Fig. 5C), implying a potential involvement of AR signaling in immune microenvironment regulation. Single-cell RNA-seq (scRNA-seq) further distinguished M1-like and M2-like macrophage subsets, and expression of the M2-like tumor-associated macrophage (TAM) markers CD206 and CD163 was enriched in tumors with high AR expression (Fig. 5D and Fig. S5C). Meanwhile, quantitative real-time polymerase chain reaction (qRT-PCR) and immunohistochemistry (IHC) analyses of tumor tissues from the control and D&E-treated groups in vivo revealed a clear reduction in M2-like TAM infiltration after D&E treatment (Fig. 5E to G). Collectively, these results suggest that AR signaling may modulate the PCa immune microenvironment through effects on TAM polarization, and that D&E treatment may partially counteract M2-like TAM infiltration. Previous studies have shown that M2-like TAMs can secrete SPP1 to promote cancer progression [32]. Among the 166 putative targets shared by Qinghao and Aiye, SPP1 was assigned to functional module 4; however, single-cell mapping revealed that SPP1 was highly expressed in myeloid cells, pointing to a close connection with module 2 (Fig. 5H and Fig. S5D). This cross-module divergence between the expression pattern and functional annotation of SPP1 prompted our particular attention. AR and SPP1 showed a positive correlation in expression in PCa tissues (Fig. 5I and J). Spatial transcriptomic analysis further revealed prominent infiltration of M2-like macrophages in PCa tissues, with these regions located in close proximity to areas of AR expression. In addition, the distribution of SPP1 substantially overlapped with that of CD206, consistent with the possibility that SPP1 is derived from M2-like macrophages (Fig. 5K).

An indirect coculture system was then established using Transwell chambers. TAMs represent the macrophage phenotype most closely linked to tumor cells within the TME, we first evaluated the ability of PCa cells to influence M2 polarization of THP-1 cells that had been programmed ex vivo toward an M1-like phenotype (Fig. 5L). Flow cytometric analysis of M1-polarized THP-1 cells from the indirect coculture showed that D&E treatment markedly reduced CD206 expression compared with the untreated group (Fig. 5M and Fig. S5E). In parallel, the mRNA levels of CD206, CD163, and SPP1 in M1-polarized THP-1 cells were preferentially up-regulated by coculture with WT or shNC cells (Fig. 5N and Fig. S5F). In line with previous reports, these findings suggest that tumor cells with high AR expression may drive macrophages toward an M2-like state, thereby contributing to the establishment of an immunologically “cold” TME [33,34].

M2-polarized THP-1 cells exposed to D&E showed reduced expression of CD163, CD206, and SPP1 (Fig. 5O). In addition, in vivo experiments showed a decrease in SPP1 expression within tumors from D&E-treated mice, accompanied by a reduction in M2-like macrophage infiltration (Fig. 5P). Molecular docking analysis further raised the possibility that SPP1 may represent a shared target of D&E (Fig. S5G). These findings suggest that D&E may suppress SPP1 secretion by macrophages.

### D&E suppresses PCa proliferation by modulating the SPP1–CD44 axis

**Fig. 6.**
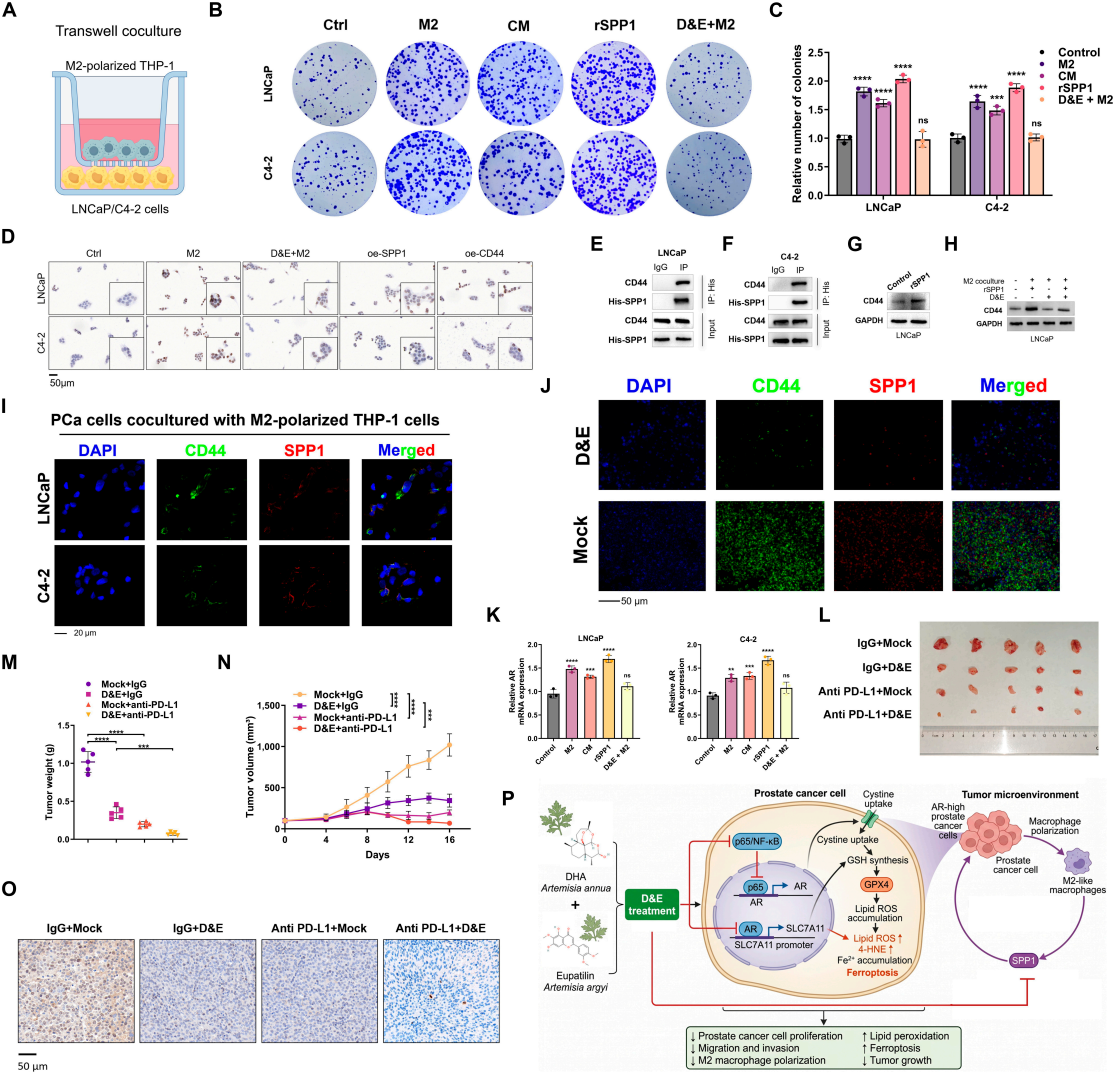
D&E suppresses PCa proliferation and metastasis by inhibiting SPP1–CD44 signaling. (A) Schematic illustration of the Transwell coculture system using ex vivo induced M2-polarized THP-1 cells together with LNCaP or C4-2 cells. (B and C) Colony formation images (B) and quantitative analysis (C) of LNCaP and C4-2 cells after treatments, including coculture with M2-like THP-1 macrophages, M2 macrophage-conditioned medium, recombinant human SPP1, or D&E-pretreated M2 macrophages. (D) qRT-PCR analysis of AR mRNA expression in LNCaP and C4-2 cells under the same condition in (B). (E and F) Co-IP assays were performed in M1-polarized THP-1 cells using an anti-His antibody. (G) Western blotting was used to evaluate CD44 expression in LNCaP cells following treatment with or without recombinant human SPP1. (H) Protein level of CD44 in LNCaP cells under the indicated coculture and treatment conditions, including M2-like macrophages, recombinant human SPP1, and high-dose D&E. (I) Immunofluorescence of LNCaP and C4-2 cells with His (green), SPP1 (red), and DAPI (blue). Scale bar, 20 μm. (J) Representative immunofluorescence images showing CD44 and SPP1 expression in tumor tissues from LNCaP xenografts in C57BL/6 mice treated with or without D&E. Scale bar, 50 μm. (K) KI-67 assay showed the proliferation of LNCaP and C4-2 cells after the following treatments: coculture with M2-like macrophages, coculture with M2-like macrophages pretreated with high-dose D&E, and rescue experiments via overexpression of SPP1 in THP-1 cells or overexpression of CD44 in LNCaP/C4-2 cells. (K to N) C57BL/6 mice (*n* = 5 per group) were subcutaneously inoculated with TRAMP-C2 cells. Mice were intraperitoneally administered D&E for 16 consecutive days, and treated with IgG or 200 μg of anti-PD-L1. Mice were sacrificed on day 17, and tumors were harvested (L). Tumor weight (M) and volume (N) were measured. IHC was performed to compare the differences in Ki-67 expression among groups (O). (P) The schematic mechanism diagram in this research. All data are shown as mean ± SD, *P < 0.05, **P < 0.01, ***P < 0.001, ****P < 0.0001, n.s., not significant vs. control group, Student *t* test. Multiple groups: one-way ANOVA + Tukey’s post hoc test. Three independent biological experiments were performed.

Molecular docking and target analysis suggested that SPP1 may be one of the targets of D&E treatment (Fig. S6A), raising the possibility that D&E might also suppress the malignant behavior of PCa cells through an SPP1-initiated signaling route. We found that coculture with M2-like THP-1 cells, or treatment with recombinant human SPP1, enhanced malignant phenotypes and increased AR expression in LNCaP and C4-2 cells (Fig. 6A to D). By contrast, M2-like macrophages exposed to D&E largely lost their tumor-promoting capacity. Previous studies have shown that SPP1 transduces signals through binding to its receptors, including CD44 [32,35]. It is therefore plausible that SPP1 secreted by M2-like macrophages promotes tumor cell proliferation through receptor engagement and downstream signaling. To test this possibility, we performed additional analyses to determine whether tumor-derived CD44 could interact with SPP1 produced by M2-polarized THP-1 cells. Co-immunoprecipitation (co-IP) assays showed that His-tagged SPP1 secreted by M2-polarized THP-1 cells interacted with CD44 in PCa cells (Fig. 6E and F). In addition, recombinant SPP1 increased CD44 expression in human PCa cells (Fig. 6G and Fig. S6B), and in D&E-treated tumor cells, recombinant SPP1 restored the reduction in CD44 expression (Fig. 6H and Fig. S6C). Immunofluorescence further demonstrated colocalization of His-tagged SPP1 and CD44 in vitro and vivo models (Fig. 6I and J), and expression of both SPP1 and CD44 was reduced following D&E treatment. Notably, in M2-like THP-1/LNCaP and M2-like THP-1/C4-2 coculture systems, overexpression of CD44 in tumor cells or SPP1 in THP-1 cells counteracted the D&E-induced changes in Ki-67 caused by treatment of M2-like THP-1 cells with D&E (Fig. 6K). These findings suggest that D&E may also suppress PCa growth by inhibiting SPP1–CD44 signaling within the TME.

Considering the emerging importance of TAMs in prostate tumor progression and their promise as treatment targets [36], we further investigated whether D&E could also enhance the efficacy of anti-PD-L1 therapy in PCa. To assess the in vivo therapeutic effect, we established a subcutaneous prostate tumor model by implanting TRAMP-C2 cells into C57BL/6 mice. The mice were then treated with anti-PD-L1 alone or together with D&E. Notably, the dual-treatment strategy (D&E + anti PD-L1) exhibited a stronger inhibitory effect on tumor growth than single-agent treatment (Fig. 6L to N). Moreover, IHC results showed a clear decline in Ki-67 expression in tumor tissues from mice receiving the combination therapy (Fig. 6O). Overall, we illustrated the putative mechanism underlying the effects of D&E (Fig. 6P).

## Discussion

PCa remains one of the leading genitourinary cancers in the male population [1]. Natural products and their derivatives have emerged as promising candidates for PCa therapy. *A. annua L.* and *A. argyi* Levl. et Van., 2 representative medicinal plants of the *Asteraceae* family, have attracted increasing attention because of their potential antitumor activities [37]. We demonstrate that the combination of DHA (derived from *A. annua*) and eupatilin (derived from *A. argyi*), hereafter referred to as D&E, functions not simply as an additive mixture of natural products but rather as a mechanism-guided combinatorial strategy. By simultaneously targeting metabolic and microenvironmental vulnerabilities in PCa, D&E exhibits robust antitumor activity while maintaining a favorable safety profile with minimal in vivo toxicity.

Mechanistically, ferroptosis appears to constitute a major contributing mechanism of the cytotoxic effects exerted by the D&E combination on PCa cells. Transcriptomic profiling, together with rescue assays using selective inhibitors of regulated cell death, indicated that D&E treatment induced oxidative stress-related and ferroptosis-associated changes. PCa cells are typically reliant on metabolic rewiring and antioxidant defense programs to survive inhibition of AR signaling [38,39]. In this context, the ferroptosis-inducing capacity of D&E may represent a therapeutically actionable vulnerability [40]. As an important derivative of a bioactive constituent from traditional Chinese medicine, DHA has been shown in multiple studies to exert anticancer activity both in vitro and in vivo [41]. Consistent with our observations, DHA has also been reported to induce both ferroptosis and apoptosis in head and neck cancer, thereby suppressing cellular proliferation [42]. In the combined D&E regimen observed here, moreover, the addition of eupatilin may further potentiate DHA-driven ferroptosis in PCa relative to DHA monotherapy, although direct evidence supporting a ferroptosis-inducing role for eupatilin in previous cancer studies remains relatively limited.

A review of the recent studies suggests that AR has been relatively underexplored as a direct target of DHA. In the present study, we therefore focused directly on AR suppression under D&E combination treatment. Although this strategy may have obscured a fuller delineation of the distinct mechanisms by which each single agent modulates the AR axis, it also opened a therapeutic perspective that is unique to the combination setting. AR has long been recognized as a central driver of proliferative and survival signaling in PCa while also serving as a central therapeutic target in this disease [43]. In line with previous observations [44], we confirmed direct occupancy of SLC7A11 by AR to promote its transcription. Through this mechanism, AR functions as a central regulator of ferroptosis resistance. Our preliminary data further indicate that AR is one of the major targets of D&E, which appears to disrupt this tumor-protective axis by suppressing the expression of SLC7A11, thereby impairing cystine utilization and weakening the antioxidant capacity of tumor cells [45].

Indeed, compounds derived from traditional Chinese medicine often suppress tumor progression through diverse mechanistic axes [46], making it unsurprising that D&E may exert concomitant antitumor effects through additional pathways. Through bioinformatic analyses, we found that D&E suppresses the NF-κB subunit P65, which notably serves as an upstream transcription factor of AR. This finding preliminarily suggests a dual regulatory effect of D&E on both P65 and AR. Through this mechanism, D&E may not only attenuate AR signaling, but also constrain NF-κB-driven maintenance of AR transcription, a feature frequently observed in androgen-independent PCa [47]. Distinct from previous studies, however, we further sought to explore the effects of D&E within a broader context by leveraging single-cell analyses. Beyond its impact on tumor-intrinsic signaling pathways, D&E also appears to exert substantial effects on the TME. An immunosuppressive TME is now recognized as an important basis for PCa progression and therapeutic resistance [36]. Our findings provide preliminary support for a reciprocal loop between prostate tumor cells and macrophages. Specifically, PCa cells promoted M2-like polarization of THP-1 cells, while M2-polarized THP-1 cells, in turn, enhanced the proliferation of PCa cells, thereby establishing a protumorigenic feedback circuit. Within this circuit, M2-like THP-1 cells secrete SPP1, which interacts with the CD44 receptor on tumor cells to reinforce malignant phenotypes [48,49] and augment AR signaling. Notably, treatment of M2-like THP-1 cells with D&E alone altered their M2-like phenotype, reduced SPP1 secretion, and lowered their ability to promote PCa cell proliferation through the SPP1–CD44 axis. Recent studies have increasingly established SPP1 as a protumorigenic factor closely linked to multiple TMEs [50], with particular relevance in PCa to research themes centered on the AR axis and bone metastasis [51]. Collectively, these findings indicate that D&E not only targets tumor-intrinsic survival pathways, but also disrupts key signaling interactions within the TME. Thus, the antitumor activity of D&E in PCa does not appear to rely solely on dual suppression of a single regulatory axis, but may also be achieved through remodeling of the tumor immune microenvironment.

Functional analysis of the shared targets of *A. annua* and *A. argyi* suggested that D&E combination treatment may influence the tumor immune microenvironment. Indeed, the findings described above had already indicated that D&E suppresses the AR axis, a result that is consistent with the current mainstream pharmacological strategies for PCa in the clinical setting. Beyond this, we further explored the application of D&E in combination with anti-PD-L1 therapy in PCa and observed a cooperative therapeutic effect between D&E and immune checkpoint blockade [52]. In vivo experiments demonstrated that the combination of D&E with anti-PD-L1 therapy significantly restrained tumor progression. Although anti-PD-L1 treatment is not a mainstream therapeutic modality for PCa in current clinical practice, an increasing number of studies have pointed to the potential of combination-based strategies [53], and D&E may represent one such traditional Chinese medicine-derived candidate that can be effectively paired with this approach. These findings provide mechanistic insight into the anticancer potential of *Artemisia*-derived compounds.

Although the inhibitory effects of D&E on key oncogenic factors such as P65, AR, and SPP1 underscore its therapeutic potential in PCa, several limitations should be acknowledged. First, our mechanistic framework was primarily established in AR-positive PCa models instead of the PDX model, and the therapeutic efficacy of D&E in AR-low, AR-null, or treatment-emergent neuroendocrine PCa requires further investigation [54]. Second, although multiomic analyses and molecular docking suggest interactions between D&E components and the P65/AR axis, additional structural studies and in vivo genetic validation will be necessary to confirm these target engagements. Moreover, the contribution of the SPP1–CD44 signaling axis to the observed enhanced antitumor effect between D&E and immune checkpoint blockade warrants further exploration, ideally using macrophage-specific SPP1 knockout models [55]. Finally, clinical translation of this mechanism-guided combination will require comprehensive pharmacokinetic and pharmacodynamic characterization, optimization of dosing strategies, and rigorous formulation standardization [56]. Addressing these issues will be essential for advancing D&E from a promising preclinical concept toward a clinically viable therapeutic approach. Given the clinical importance of AR splice variants, especially AR-V7, in CRPC, it is worth noting that AR-V7 lacks the ligand-binding domain and is therefore not effectively targeted by conventional AR antagonists [4]. Since AR-V7 arises from the AR gene locus, and NF-κB/p65 signaling has been reported to promote both AR and AR-variant expression, inhibition of the p65–AR transcriptional axis by D&E may theoretically also affect AR-V7-associated signaling [57]. However, as AR-V7 was not directly assessed in the current study, this possibility remains speculative and warrants future validation in AR-V7-positive models.

## Materials and Methods

### Analysis of public RNA-seq datasets and RNA-seq of PCa cells after D&E treatment

Transcriptomic profiles coupled with matched clinical metadata for the PRAD cohort were retrieved from TCGA (https://www.cancer.gov/ccg/research/genome-sequencing/tcga). Additional RNA-seq datasets were obtained from the GEO database (https://www.ncbi.nlm.nih.gov/gds/?term=), including GSE17044, GSE31978, and GSE63693. For public datasets that had not been log-transformed, log2 transformation was first applied. To identify differentially expressed genes, the “limma” package in R (version 3.40.2) was used. To elucidate the biological significance of the identified genes, functional annotation and pathway overrepresentation analyses—incorporating both GO terms and KEGG pathways—were executed via the *clusterProfiler* package within the R environment. LNCaP cells were treated with or without D&E, and total RNA was then extracted. The RNA-seq workflow included assessment of RNA concentration and integrity, cDNA library construction, and sequencing on the Illumina NovaSeq platform. After the removal of low-quality reads and adaptor contamination, the resulting clean reads were aligned to the human reference genome (GRCh38/hg38). Subsequently, the isolated RNA was reverse-transcribed into cDNA using a reverse transcription kit (Cat. No. RR036A, Takara, Japan), followed by amplification and detection on a qRT-PCR system using SYBR Green Premix (Cat. No. RR420A, Takara, Japan).

### Single-cell and spatial transcriptomic data

Datasets for single-cell and spatial transcriptomics were obtained from the GEO with accession numbers GSE141445, GSE153892, and GSE230282. Raw scRNA-seq count matrices underwent preprocessing and doublet removal utilizing the *Seurat* (v5.3.1) and *scDblFinder* (v1.23.9) packages. Cells were strictly filtered based on quality control metrics, retaining those with expressed gene features (nFeature_RNA) between 200 and 6,000, alongside a mitochondrial transcript proportion below 15%. Following *SCTransform* normalization, principal component analysis (PCA) was applied to the expression matrices. Unsupervised clustering was executed via a shared nearest neighbor (SNN) graph approach. Broad cell lineages were manually assigned based on well-established canonical marker genes. To comprehensively delineate TME heterogeneity, the epithelial and myeloid populations were isolated and subjected to independent, high-resolution reclustering workflows. Additionally, an in silico virtual knockout of the AR was simulated utilizing *scTenifoldKnk* (v1.0.3). Ferroptosis activity at single-cell resolution was quantified via *AUCell* (v1.30.1), referencing signature gene sets curated from the FerrDb database (http://www.zhounan.org/ferrdb/current/). To eliminate potential sequencing-depth biases, the computed AUCell scores were corrected via a linear regression model and subsequently standardized into *Z*-scores. Finally, to validate intercellular interactions in situ, 10x Genomics Visium spatial transcriptomic data were integrated into our analytical pipeline. Following *SCTransform* normalization of the spatial expression matrices, cell-type deconvolution was performed employing the *CARD* algorithm (v1.1), using our comprehensively annotated scRNA-seq dataset as the spatial reference map.

### Immune infiltration analysis

To evaluate immune cell infiltration, we analyzed the GSE32269 dataset from the GEO repository. Following variance stabilization of the gene expression profiles via *voom* transformation, data wrangling and visualization were executed utilizing a suite of R packages (*limma*, *e1071*, *parallel*, *preprocessCore*, *ggplot2*, *ggpubr*, *reshape2*, *RColorBrewer*, and *pheatmap*). Deconvolution of the immune microenvironment was achieved through linear support vector regression (SVR). Briefly, the SVR algorithms were trained across 3 distinct v parameters (0.25, 0.5, and 0.75), and the optimal model was determined based on the minimum root mean square error (RMSE); subsequently, negative weight coefficients were constrained to zero prior to normalization. Statistical significance was established by calculating empirical *P* values derived from 1,000 permutations. Prior to deconvolution, the reference matrix underwent *Z*-score standardization, while the mixture matrix was quantile-normalized. Downstream analyses were strictly restricted to samples demonstrating a deconvolution *P* < 0.05. Furthermore, we assessed the Spearman rank correlation between the expression of AR and the estimated abundances of infiltrating immune cells. Data manipulation and correlation profiling were facilitated by the *limma*, *dplyr*, *tidyverse*, and *ggplot2* packages; the resulting associations were stratified by both statistical significance (*P* < 0.05) and the magnitude of the correlation coefficient (<0.2, 0.2 to 0.4, 0.4 to 0.6, and ≥0.6). Finally, applying an identical statistical framework, we evaluated the correlation between AR expression and the enrichment metric of the “GOBP_IMMUNE_RESPONSE_TO_TUMOR_CELL” signature, retrieved from the Molecular Signatures Database (MSigDB).

### Drug target screening

Potential active compounds were retrieved from the TCMSP database by searching the terms *A. argyi* (“Aiye”) and *A. annua* (“Qinghao”). Compounds meeting the screening criteria of oral bioavailability of at least 30% and drug-likeness of at least 0.18 were considered candidate constituents, and their predicted therapeutic targets were gathered for further investigation.

### Molecular docking

Coordinates in 3 dimensions for the target proteins were sourced from the Protein Data Bank (https://www.rcsb.org/). To ensure docking fidelity, candidate structures were subjected to stringent filters: we strictly utilized human-derived proteins and prioritized high-resolution x-ray diffraction models (≤2.5 Å); optimal models cocrystallized with native ligands were preferentially selected, with a distinct preference for binding pockets comprising unmodified and nonphosphorylated amino acid residues. The corresponding structural data for the key small-molecule active compounds were retrieved from the PubChem repository. Prior to simulation, macromolecular receptors were prepared utilizing PyMOL (version 2.4.0) through the systematic removal of solvent molecules, the addition of polar hydrogens, and the excision of original cocrystallized ligands. In silico molecular docking was subsequently executed employing AutoDock Vina. Thermodynamic binding feasibility was evaluated based on affinity scores; an overall negative binding free energy denoted spontaneous interaction, while a threshold of <−5 kcal/mol was established to indicate highly favorable receptor–ligand complexation. Finally, docking conformations exhibiting the most optimal binding affinities were extracted and visualized via PyMOL 2.4.0.

### Bioinformatics analysis using online databases

GEPIA3 [58] (https://gepia3.bioinfoliu.com/) was used to evaluate the correlations between genes. TIMER2 analysis was performed to determine whether AR expression was associated with macrophage infiltration in TCGA-PRAD. TF-Target Finder [59] (https://jingle.shinyapps.io/TF_Target_Finder/) was used to predict potential transcription factors. From the Cistrome database [60] (http://dbtoolkit.cistrome.org/), we obtained ChIP-seq data and corresponding meta-analysis results for AR binding to SLC7A11 and P65 binding to AR. The JASPAR database (https://jaspar.elixir.no/) was used to predict transcription factor binding sites, which were further visualized using the UCSC (https://genome.ucsc.edu/).

### Sources and culture of cells and animals

The human PCa cell line LNCaP (RRID: SCSP-5021) and the human monocytic cell line THP-1 (cell bank number: SCSP-567) were purchased from the Cell Bank of the Chinese Academy of Sciences (Shanghai, China). The human PCa cell line C4-2 (CRL-3314) and the murine PCa cell line TRAMP-C2 (CRL-2731) were obtained from the American Type Culture Collection. All cells were cultured in RPMI-1640 medium (Cat. No. SH30809.01, Cytiva, China) or Dulbecco's modified Eagle medium (DMEM, Cat. No. SH30243.02, Cytiva, China) supplemented with 10% fetal bovine serum (Cat. No. 16000044, Thermo Fisher Scientific, USA) and 1% penicillin–streptomycin (Cat. No. 15140122, Gibco, USA) in a humidified incubator at 37 °C with 5% CO_2_. In some experiments, androgen-deprived culture conditions were established as previously described [61]. For ferroptosis-related experiments, some cells were cultured in cystine-low medium. Authentication of all cell lines was performed via short tandem repeat (STR) profiling, and they were found to be free from mycoplasma contamination. The source of the 6-week-old male BALB/c nude mice and C57BL/6 mice was Super-B&K Laboratory Animal Corp. Ltd. (Shanghai, China) and housed under specific pathogen-free conditions. All experiments involving animals followed the approved institutional guidelines of the Experimental Animal Ethics Committee of Chongqing Medical University.

### CDX models

To establish subcutaneous xenograft models, viable LNCaP or C4-2 cells (5 × 10^6^ per BALB/c nude mouse) and TRAMP-C2 cells (5 × 10^6^ per C57BL/6 mouse) were suspended in 100 μl of a 1:1 mixture comprising serum-free basal medium and Matrigel, and subsequently inoculated into the right inguinal flanks of the animals.

Upon tumors attaining an average volume of approximately 100 mm^3^ (typically 1 week post-engraftment), the mice were randomized into vehicle control or D&E treatment cohorts. Subjects in the D&E cohort received daily intraperitoneal (i.p.) administration of DHA (25 mg/kg in 200 μl) alongside concurrent i.p. injections of eupatilin (10 mg/kg in 80 μl) every other day for 3 weeks. Tumor dimensions were monitored at regular intervals, with volumes approximated utilizing the standard ellipsoid formula: *V* = (length × width^2^)/2. Following 21 days of continuous treatment, the animals were euthanized on day 22 to facilitate the surgical excision and gravimetric assessment of the tumor tissues. For the immunotherapy combination assays, a designated subset of mice was additionally treated with an anti-PD-L1 monoclonal antibody (clone 10F.9G2, Bio X Cell, USA; 100 μg/mouse, i.p., once every 3 days), with terminal tumor harvesting conducted on day 17. Furthermore, to simulate in vivo androgen deprivation, a separate cohort of BALB/c nude mice bearing C4-2 xenografts underwent surgical castration (bilateral orchiectomy). When the tumor volumes reached ~200 mm^3^, these animals were randomized into either a sham-operated control arm or the surgical castration arm. Neoplastic tissues from these models were systematically harvested at designated time points across a 2-week longitudinal window to support subsequent molecular analyses, with 5 biological replicates (*n* = 5) included per condition.

### DHA and eupatilin

DHA (Cat. No. S2290) and eupatilin (Cat. No. S3846) were sourced from Selleck Chemicals. DHA is an active derivative of *A. annua*, whereas eupatilin is a flavonoid monomer derived from plants of the *Artemisia* genus. Stock solutions of both compounds were stored at −20 °C. An equal volume of DMSO (Cat. No. D2650, Sigma-Aldrich, USA) was added to the control group to ensure the same final concentration in all groups. According to the experimental design, a DHA monotherapy group, a eupatilin monotherapy group, and a combined treatment group (D&E) were established. The mixing ratio of D&E was determined based on the respective IC_50_ values of DHA and eupatilin in the tumor cell lines, with a DHA:eupatilin ratio of approximately 19:6 in LNCaP cells and 6:10 in C4-2 cells (total concentration method). Based on the IC_50_ values obtained for the D&E combination at the above ratios, different concentration gradients were set for subsequent experiments in LNCaP and C4-2 cells. Specifically, 0.25 × D&E IC_50_, 0.5 × D&E IC_50_, and 1 × D&E IC_50_ were defined as the low-, medium-, and high-dose concentrations, respectively. Drug interaction was assessed by Bliss independence and Chou–Talalay analyses using fixed-ratio DHA/eupatilin combination data. Bliss-predicted effects were calculated from single-agent inhibitory effects, and Bliss excess was defined as observed minus predicted inhibition. For Chou–Talalay analysis, Fa values were derived from cell viability, and CI values were calculated from the fitted single-agent dose–response curves. CI < 1 was considered synergistic.

### Macrophage polarization and indirect coculture

THP-1 cells were stimulated with phorbol 12-myristate 13-acetate (PMA, CAS No. 16561-29-8; Selleck, USA) for 24 h to induce differentiation into adherent macrophage-like cells. After PMA withdrawal, the cells were further cultured to obtain M0-like macrophages. These cells were then polarized to the M1-like/M2-like phenotype by LPS (Cat. No. 00-4976-93; Thermo Fisher, USA, 24 h) or IL-4 (Cat. No. 200-04-1MG; Thermo Fisher, USA, 24 h), respectively. An indirect coculture model of PCa cells and polarized macrophages was established using Transwell inserts with a pore size of 0.4 μm (Cat. No. 14312; LABSELECT, China). After coculture, macrophages or tumor cells in the lower chamber were collected separately to examine changes in the expression of CD206, SPP1, AR, and other related molecules. For drug intervention experiments, D&E treatment was added after macrophages had completed M2-like polarization to evaluate its effects on M2-like polarization and SPP1 secretion. This method was adapted with reference to previous studies on macrophage polarization and tumor coculture [62]**.**

### Cell transfection and stable cell line generation

Lentiviral vectors carrying the full-length coding sequences of RELA (NM_021975.4), CD44 (NM_000610.4), and SPP1 (NM_001251830.1) were obtained from GeneCopoeia (USA) to generate stable overexpression cell lines. Viral particles were produced by cotransfecting these vectors into HEK293T cells and were subsequently used to infect PCa cells. After 48 h, cells were selected (2 μg/ml puromycin) for 7 days to establish stable cell lines. The full-length coding sequence of human AR (NM_000044.6) was amplified by PCR and subcloned into a mammalian expression vector containing an N-terminal FLAG tag using standard restriction enzyme digestion and ligation procedures. To construct the HA-SLC7A11 expression vector, SLC7A11 cDNA was amplified by PCR using the following primers (SLC7A11-F: 5​′-C​GGT​GGA​CCA​GTG​CAG​AAA​GCT​GTG​TGT​GTCCACC-3′; SLC7A11-R: 5′-CAGCGCGCGCGGTTTTCGACAACTCCAGTAT-3′) and then subcloned into the pCMV-HA vector. To knock down AR, RELA, and SLC7A11, shRNA plasmids were purchased from Sigma-Aldrich, and the corresponding sequences are listed in Table S2. According to the manufacturer’s instructions, shRNA plasmids were transfected into PCa cells using Lipofectamine 3000 (Cat. No. L3000015; Invitrogen, USA). Knockdown efficiency was then confirmed by Western blotting.

### qRT-PCR

For transcriptomic quantification, total cellular RNA was isolated from the respective cell lines utilizing TRIzol reagent (Cat. No. 15596018, Invitrogen). To eliminate genomic DNA contamination and facilitate first-strand cDNA synthesis, the PrimeScript RT reagent kit was employed. Subsequent qRT-PCR assays were executed utilizing TB Green Premix Ex Taq (Tli RNase H Plus) alongside target-specific oligonucleotides. The precise primer sequences deployed for these amplification cycles are comprehensively cataloged in Table S2.

### Western blotting analysis

Whole-cell proteins were extracted with standard lysis buffer, separated by sodium dodecyl sulfate–polyacrylamide gel electrophoresis (SDS-PAGE), and transferred to polyvinylidene fluoride (PVDF) membranes (Cat. No. 1620177, Bio-Rad, USA). After incubation with primary antibodies at 4 °C overnight, membranes were exposed to horseradish peroxidase (HRP)-conjugated anti-rabbit (Cat. No. ab6721, Abcam, USA) or anti-mouse (Cat. No. ab6728, Abcam, USA) secondary antibodies. Protein bands were detected using an ECL kit (Cat. No. 1705060, Bio-Rad, USA). We also separately extracted nuclear and cytoplasmic/membrane protein fractions for Western blot analysis to assess changes in protein subcellular localization. Briefly, after lysis with cytoplasmic lysis buffer, the lysates were centrifuged at low speed (800 × *g*) to separate the nuclear pellet from the supernatant. The supernatant was collected as the cytoplasmic/membrane fraction. The nuclear pellet was gently washed with phosphate-buffered saline (PBS) or cytoplasmic lysis buffer, followed by complete lysis in high-salt nuclear extraction buffer with incubation on ice and intermittent vortexing. After high-speed centrifugation, the supernatant was collected as the nuclear protein fraction. Detailed antibody information is provided in Table S2.

### CCK-8 assay and IC_50_ determination

CCK-8 assays (Cat. No. K1018, APExBIO, USA) were used to measure cell proliferation and viability. Cells were seeded in 96-well plates at a density of 1 × 10^3^ cells/well, then treated with different doses of DHA, eupatilin, or D&E for 48 h. After 2 h OF incubation with CCK-8 reagent, absorbance at 450 nm was recorded, and IC_50_ values were calculated (GraphPad Prism 10.1.2).

### Colony formation assay

To evaluate clonogenic capacity, single-cell suspensions were plated in 6-well receptacles at an initial density of 1,000 cells per well. Following exposure to the designated interventions, the cells were maintained for a 14-day growth period, with the nutrient medium refreshed every 3 to 4 days. Macroscopic colonies were subsequently immobilized using 4% paraformaldehyde (Cat. No. P0099, Beyotime, China) and visualized via a 20-min staining protocol with 1% crystal violet (Cat. No. C0121, Beyotime, China). After a gentle rinse with tap water and complete air-drying, colony enumeration and areal quantification were executed utilizing ImageJ software.

### EdU assay

PCa cells in the logarithmic growth phase under different pretreatment conditions were seeded into 6-well plates containing sterile glass coverslips at a density of 1 × 10^5^ cells/ml. After overnight attachment, cells were cultured until they reached more than 80% confluence and then treated with D&E. 5-Ethynyl-2′-deoxyuridine (EdU) incorporation was detected using an EdU assay kit according to the manufacturer’s instructions (Beyotime, C0075S). Briefly, EdU working solution was added to the culture medium at a final concentration of 10 μM, and the cells were incubated at 37 °C in a humidified incubator with 5% CO₂ for 2 h. After incubation, the cells were washed with PBS and fixed with 4% paraformaldehyde at room temperature. Subsequently, the cells were washed with PBS and permeabilized with 1 ml of 0.3% Triton X-100 permeabilization buffer for 10 to 15 min (Beyotime, P0096). The cells were then incubated with the Click-iT reaction mixture for 30 min at room temperature in the dark. After washing with PBS, the nuclei were counterstained with 4′,6-diamidino-2-phenylindole (DAPI) for 5 to 10 min.

### Wound-healing assay

PCa cells in the logarithmic growth phase under different pretreatment conditions were seeded into 6-well plates and cultured overnight. When the cells reached approximately 90% to 100% confluence, a straight scratch wound was generated in the cell monolayer using a sterile 200-μl pipette tip. Detached cells were gently removed by washing with PBS, and the cells were then cultured in serum-free medium with or without D&E to minimize the influence of cell proliferation. Images of the wound area were captured after scratching using an inverted microscope.

### Ferroptosis-related assays

Ferroptosis inhibitor assay: PCa cells were treated with D&E in combination with the ferroptosis inhibitors DFO (10 μM, Cat. No. D9533, Sigma-Aldrich, USA), Fer-1 (1 μM, Cat. No. A4371, APEXBIO, USA), and CPX (10 μM, Cat. No. B2087, APEXBIO, USA). Other cell death inhibitors used in this study included the necroptosis inhibitor necrostatin-1 (10 μM, Cat. No. A4213, APEXBIO, USA), the pyroptosis-related inhibitors MCC950 (10 μM, Cat. No. C3780, APEXBIO, USA) and VX-765 (10 μM, Cat. No. A8238, APEXBIO, USA), the autophagy inhibitor 3-methyladenine (5 mM, Cat. No. A8353, APEXBIO, USA), and the apoptosis inhibitor z-VAD-FMK (10 μM, Cat. No. A1902, APEXBIO, USA). After 48 h, cell viability was assessed using a CCK-8 assay.

LDH release assay: LDH Cytotoxicity Assay Kit (Cat. No. G1610-100T, Servicebio, China) was employed in this section of the experiment. Cells were seeded in 6-well plates for 48 h. Lactate in the culture medium was oxidized through an enzymatic reaction to generate a colored product, and absorbance was measured at 570 nm. The percentage of cytotoxicity was calculated as follows: (absorbance of treated group − background control) / (maximum release control − background control) × 100%.

Lipid peroxide detection: Cells were incubated in 60-mm dishes and stained with 5 μmol/l BODIPY 581/591 C11 dye (Cat. No. 27086, Cayman Chemical, USA) for 25 min. The cells were then washed with PBS, trypsinized, and analyzed by flow cytometry using the fluorescein isothiocyanate (FITC) channel to determine intracellular lipid peroxide levels (lipid ROS).

MDA assay: Intracellular MDA levels were determined using an MDA Assay Kit (Cat. No. S0131, Beyotime, China) to reflect the extent of lipid peroxidation.

GSH measurement: For this part of the experiment, we used the GSH Assay Kit (Cat. No. 703002, Cayman Chemical, USA). In the experiment, cell lysates were collected for analysis, and the detectable signal generated by the reaction system reflected the GSH content in the samples. The obtained GSH values were further normalized based on the total protein concentration measured by the bicinchoninic acid (BCA) method.

TEM: PCa cells subjected to D&E treatments were collected and fixed in 2.5% glutaraldehyde in phosphate buffer for 2 h at 4 °C, followed by post-fixation in 1% osmium tetroxide for 2 h. Samples were dehydrated through a graded ethanol series, transitioned with acetone, and embedded in resin. Ultrathin sections (50 to 60 nm) were prepared and stained with 2% uranyl acetate and lead citrate. Images were acquired using a transmission electron microscope (JEM-1011, JEOL, Japan). Mitochondrial ultrastructural changes, including mitochondrial shrinkage, increased electron density, and reduction or disruption of cristae, were evaluated as ferroptosis-associated morphological features.

### Ki-67 assay

To evaluate cellular proliferation, specimens were probed with an anti-Ki-67 antibody (ab16667; Abcam, Cambridge, UK). For in vitro immunofluorescence analysis, cultured cells subjected to designated treatments were immobilized in 4% paraformaldehyde for 15 min, permeabilized utilizing 0.1% Triton X-100 (T9284, Sigma-Aldrich, USA) for 10 min, and subsequently blocked with 5% bovine serum albumin (BSA) at ambient temperature for 30 min. The samples were then exposed to the primary Ki-67 antibody and maintained overnight at 4 °C. Following sequential PBS washes, a fluorophore-conjugated secondary antibody was introduced for a 1-h dark incubation at room temperature, concluding with DAPI counterstaining to visualize nuclei. Fluorescent signals were ultimately recorded utilizing an epifluorescence microscope. For in vivo proliferation assessment via IHC, excised murine tumor xenografts were fixed in 4% paraformaldehyde, embedded in paraffin wax, and sliced into 4-μm-thick sections. Tissue slides underwent deparaffinization in xylene and graded alcohol rehydration, followed by heat-induced epitope retrieval in a pH 6.0 citrate buffer. Endogenous peroxidase quenching was achieved using 3% hydrogen peroxide, and nonspecific binding sites were masked with 5% BSA for 30 min at room temperature. The tissue sections were subsequently incubated with the primary anti-Ki-67 antibody overnight at 4 °C. Post-washing with PBS, an HRP-linked secondary antibody was applied for a 30-min room temperature incubation, and the chromogenic signal was developed utilizing a DAB substrate before hematoxylin counterstaining. Final histopathological images were acquired utilizing a bright-field light microscope.

### Flow cytometry analysis

Cells receiving the indicated treatments were collected. Tumor cell apoptosis was measured with an Annexin V-FITC/PI apoptosis detection kit (Cat. No. CA1020, Solarbio, China), with a 15-min dark incubation. To assess phenotype polarization markers in THP-1-derived macrophages, cells were resuspended in staining buffer and incubated at 4 °C for 30 min in the dark with anti-human CD86-PE (Cat. No. ab77226, Abcam, UK) or CD206-APC (Cat. No. 550889, BD Biosciences, USA). The stained cells were then washed with PBS, fixed in 4% paraformaldehyde for 1 h at 4 °C, and subjected to flow cytometric analysis under optimized acquisition settings. FlowJo software was used for data analysis.

### IHC staining and scoring of tumor tissues

IHC assays were executed in accordance with previously established protocols [63]**.** In short, murine tumor specimens were subjected to standard fixation, paraffin embedding, and microtome sectioning. Following epitope retrieval and the quenching of endogenous peroxidase activity, slides were masked using 10% normal goat serum (Cat. No. C0265, Beyotime Biotechnology, China) prior to an overnight incubation at 4 °C with the respective primary antibodies. Tissue sections were incubated with the corresponding HRP-linked secondary antibodies. Signal development was carried out with a DAB detection system (Cat. No. SK-4105, Vector Laboratories, USA), and nuclei were then counterstained using Hematoxylin QS (Cat. No. H-3404-100, Vector Laboratories, USA). After staining, the sections were mounted with an appropriate mounting medium (Cat. No. H-5000-60, Vector Laboratories, USA). IHC results were semiquantitatively scored based on staining intensity and the proportion of positive cells. Staining intensity was assigned as 0, negative; 1, weak; 2, moderate; and 3, strong. The proportion of positively stained cells was scored as 0 for 0%, 1 for 1% to 10%, 2 for 11% to 50%, 3 for 51% to 80%, and 4 for ≥80%. A final immunoreactivity score was obtained by multiplying the intensity score by the proportion score (0 to 12).

### ChIP-qPCR

ChIP Kit (Cat. No. ab500, Abcam, UK) was used. PCa cells were fixed with formaldehyde at a final concentration of 1% for 15 min to crosslink proteins to DNA. The cells were then lysed, and chromatin was fragmented by sonication using a 10-mm probe at 50 W for 2 s per pulse for a total of 4 pulses. The immunoprecipitated protein–DNA complexes were eluted, and crosslinks were reversed at 65 °C for 16 h. The purified DNA fragments were then subjected to qPCR analysis. Input control samples were prepared from the supernatant of the sonicated lysates. Ct values for each ChIP DNA sample were normalized accordingly. Fragmented chromatin was incubated with an anti-AR antibody or an IgG control antibody, followed by DNA purification and qPCR analysis. Using the same approach, the binding of P65 to the AR region was also examined. Sequences used are listed in Table S2. The relevant genomic regions were visualized using IGV (v2.19.7).

### Co-immunoprecipitation

For co-IP, cells were grown in 6-cm dishes and lysed on ice for 30 min in IP lysis buffer after collection by scraping. The lysis buffer consisted of 20 mM Tris-HCl (pH 7.5), 150 mM NaCl, 1 mM EDTA, 1 mM EGTA, 1% Triton X-100, 2.5 mM sodium pyrophosphate, 1 mM β-glycerophosphate, 1 mM Na_3_VO_4_, 1 μg/ml leupeptin, and 1 mM phenylmethylsulfonyl fluoride (PMSF). Following centrifugation (13,000 × *g*; 15 min), the clarified lysates were incubated for 5 h at 4 °C with Protein A/G beads coupled to the indicated antibody. The resulting immune complexes were then subjected to Western blot analysis with the appropriate antibodies.

### Dual-luciferase reporter assay

Based on the predicted binding sites from the JASPAR database, pGL3 reporter constructs containing either the wild-type AR promoter sequence (AR-luc-WT) or a mutant P65-binding site sequence (AR-luc-mutant) were generated (Table S2). At 24 h post-transfection, lysates were prepared using Passive Lysis Buffer (Cat. No. E1941, Promega, USA), and reporter activity was measured with the Dual-Luciferase Reporter Assay System on a GloMax 20/20 luminometer (Promega). Relative luciferase activity was calculated after normalization to the Renilla control.

### Immunofluorescence

Immunofluorescence staining was performed on FFPE tumor tissue sections using the Opal Multiplex Fluorescent Staining Kit (Cat. No. NEL811001KT; Akoya Biosciences, USA). After deparaffinization, rehydration, and antigen retrieval, the sections were sequentially incubated with primary antibodies and visualized using the corresponding Opal fluorophores. After each round of staining, microwave treatment was performed to strip the bound antibodies. Finally, nuclei were counterstained with DAPI.

### Statistical analysis

All statistical computations and graphical representations were executed utilizing GraphPad Prism (version 10.1.2) or SPSS software (version 26.0). Quantitative results are expressed as the mean ± standard deviation (SD), derived from a minimum of 3 biologically independent replicates. To evaluate statistical significance, the Student *t* test was applied for pairwise comparisons, whereas a one-way analysis of variance (ANOVA) accompanied by Tukey’s post hoc adjustment was utilized for multigroup analyses. Associations between continuous variables were quantified employing the Pearson correlation coefficient. The thresholds for statistical significance are indicated in the figure legends.

## Data Availability

The data generated in the present study are available from the corresponding authors upon reasonable request.
