# Supplementary material for: Combined Dihydroartemisinin and Eupatilin Suppress Prostate Cancer through AR-Associated Ferroptosis and Modulation of Macrophage–Tumor Crosstalk
Source: Research (Wash D C). 2026 Jun 23;9:1331. doi: 10.34133/research.1331 (PMC13287449; doi:10.34133/research.1331)
Supplement: Supplementary 1 — Figs. S1 to S6 Tables S1 to S5 [file research.1331.f1.zip › Supplementary Materials.docx]

Supplementary Figure 1. Combined treatment with Dihydroartemisinin (DHA) and Eupatilin induces ferroptosis.

(A–B) The IC50 values of DHA (A) and eupatilin (B) in LNCaP and C4-2 cells were determined using the CCK-8 assay. (C-D) Observed and Bliss-predicted cell viability values for fixed-ratio D&E shown in LNCaP and C4-2 (C) cells. The corresponding Bliss excess values are shown in LNCaP and C4-2 (D), where positive values indicate stronger-than-additive effects. (E) Combination index (CI) values were calculated for fixed-ratio DHA and eupatilin combinations in LNCaP and C4-2 cells using the Chou–Talalay method. The fraction affected (Fa) was derived from the observed cell viability of the combination treatment. The dashed line indicates CI = 1, representing an additive effect. (F-G) Subcutaneous xenografts were generated in male nude BALB/c mice (n = 5 per group). After random grouping, mice in the D&E group received combination treatment: DHA (25 mg/kg, 200 μL) was administered intraperitoneally daily, and eupatilin (10 mg/kg, 80 μL) was administered intraperitoneally every 2 days for 3 consecutive weeks. Body weight was measured regularly during treatment (F). At day 22, the mice were euthanized, and major organs were subjected to hematoxylin and eosin staining to assess potential toxic effects (G). (H) Representative TEM images of c4-2 cells treated with vehicle control or increasing concentrations of D&E (low, medium, and high dose). Scale bars, 2 μm (upper panels) and 500 nm (lower panels). All data are shown as mean ± SD, n.s., not significant vs. control group. Experiments were repeated three times independently.

Supplementary Figure 2. Prediction and validation of AR as a therapeutic target of D&E.

(A)KEGG and GO analyses were performed based on the 166 potential targets of Artemisia annua and Artemisia argyi. (B) Venn diagram presenting the seven intersecting genes between the set of 166 predicted genes and the 233 genes downregulated in response to D&E treatment. A heatmap shows the logFC changes following D&E treatment, with purple representing lower expression levels. (C–D) After treatment of LNCaP and C4-2 cells with different doses of D&E, the mRNA and protein levels of AR. (E) Ferroptosis Marker score distribution in the “Epithelial” cell cluster was visualized by UMAP using the AUCell (v1.30.1) algorithm; a redder color indicates a higher Ferroptosis Marker score. The correlation between AR expression and Ferroptosis Marker score in the Epithelial cell cluster was analyzed using Pearson correlation test. (F-G) After 72 h of infection with lentiviruses carrying the indicated shRNAs, cells were collected for qRT-PCR analysis of KLK3 mRNA (F) and western blot detection of AR (G). All data are shown as mean ± SD, *p < 0.05, **p < 0.01, ***p< 0.001, ****p < 0.0001, n.s., not significant vs. control group. Three independent biological experiments were performed.

Supplementary Figure 3. AR binds to and induces SLC7A11 expression.

(A) Enrichment analyses of the upregulated genes from GSE17044 (R1881 treatment vs. control) and GSE63692 (R1881 vs. control), respectively. (B-C) GPX4 expression after the indicated 48 h treatments was assessed by qRT-PCR. (D-E) Western blot of GPX4 cultured in CSS medium with or without DHT (D, 10 nM) or high-dose D&E (E) for 48 h. (F) Transcription factors of SLC7A11 were predicted using TF Target Finder (https://jingle.shinyapps.io/TF_Target_Finder/) across CHEA, GTRD, ChIP_Atlas, hTFtarget, and KnockTF databases. All data are shown as mean ± SD, *p < 0.05, **p < 0.01, ***p< 0.001, ****p < 0.0001, n.s., not significant vs. control group. Three independent biological experiments were performed.

Supplementary Figure 4. NF-κB mediates D&E-induced suppression of AR.

(A) JASPAR (https://jaspar.elixir.no/) sequence analysis revealed putative P65 binding sites in the AR promoter binding site. (B) qRT-PCR analysis of AR mRNA levels of cells cultured (10% CSS / high dose of D&E) with or without RELA overexpression. (C-D) Western blotting analysis of AR and P65-related proteins in LNCaP (C) and C4-2 (D) cells under the indicated treatments (10% CSS / high dose of D&E). (E–H) Intracellular LDH levels were measured (E), and lipid peroxidation was assessed by flow cytometry (F). Harvested cells were lysed, and MDA (G) and GSH (H) levels were determined using the corresponding assay kits. (I) The Cistrome web platform was used to analyze ChIP datasets and predict transcription factors with potential binding to the AR gene region. (J-K) ChIP-qPCR and dual-luciferase reporter analyses of the interaction between P65 and AR. Under conditions of P65 overexpression, with or without high-dose D&E treatment, the binding of P65 to the AR promoter region was examined (J); under the same treatment conditions, changes in AR expression in C4-2 cells were also assessed (K). (L) GSE83860 ChIP-seq results indicated a P65-enriched peak in the AR promoter region of LNCaP cells, as shown in red. (M-N) Molecular docking analysis of DHA and Eupatilin with AR and P65. Representative docking poses are shown, with enlarged views highlighting the predicted binding modes within the ligand-binding pockets. Both DHA and Eupatilin exhibited favorable interactions with AR and P65 (M). The predicted binding energies were showed (N). Data are presented as mean ± SD. ****P < 0.0001 vs. Control; ###P < 0.001, ####P < 0.0001 vs. high group. Three independent biological experiments were performed.

Supplementary Figure 5. D&E suppresses macrophage M2 polarization and SPP1 secretion in PCa.

(A) Spatial transcriptomic maps (GSE153892) showing the distribution of CD68, MRC1, CD163, and CD86 expression in PCa tissue. Feature plots indicate the spatial localization and relative expression levels of the indicated genes across the tissue section. Red indicates high expression. (B) Screenshot from the TIMER2 web server (https://compbio.cn/timer2/) showing the association between AR and macrophages in PCa. Numbers represent Pearson correlation coefficients. (C) Violin plots of SPP1 expression between AR-high and AR-low groups in myeloid cell clusters at the single-cell level. (D) Single-cell UMAP plot displaying the expression distribution of SPP1 across all cell clusters; a redder color indicates higher expression level. (E) CD206 expression in M1-polarized THP-1 cells co-cultured with C4-2 cells with or without high-dose D&E treatment, as measured by flow cytometry. The assay was independently repeated three times. (F) Expression of the M2-associated TAM genes CD206, CD163, and SPP1 in M1-polarized THP-1 cells following co-culture with WT or shAR (#1 and #2) LNCaP cells, as determined by qRT-PCR. (G) Molecular docking analysis of DHA and Eupatilin with SPP1. Representative docking poses are shown, with enlarged views highlighting the predicted binding modes within the ligand-binding pockets. All data are shown as mean ± SD, *p < 0.05, **p < 0.01, ***p< 0.001, ****p < 0.0001, n.s., not significant vs. control group, Student’s t-test. Multiple groups: one-way ANOVA + Tukey’s post hoc test. Three independent biological experiments were performed.

Supplementary Figure 6. D&E suppresses PCa proliferation and metastasis by inhibiting SPP1–CD44 signaling.

(A) Molecular docking analysis of SPP1 with CD44. Representative docking poses are shown, with enlarged views highlighting the predicted binding modes within the ligand-binding pockets. (B) Protein expression of CD44 in C4-2 cells treated with or without recombinant human SPP1 was evaluated by Western blotting. (C) Western blotting was used to detect CD44 in C4-2 cells cultured under the indicated conditions, including the presence or absence of M2-like macrophages, recombinant human SPP1, and high-dose D&E. n = 3 biologically independent samples per group.
