# Supplementary material for: Combined Dihydroartemisinin and Eupatilin Suppress Prostate Cancer through AR-Associated Ferroptosis and Modulation of Macrophage–Tumor Crosstalk
Source: Research (Wash D C). 2026 Jun 23;9:1331. doi: 10.34133/research.1331 (PMC13287449; doi:10.34133/research.1331)
Supplement: Supplementary 1 — Figs. S1 to S6 Tables S1 to S5 [file research.1331.f1.zip › Supplementary Table1-2.docx]

| **TableS1：The antibodies used in this study are listed below.** | | |
| --- | --- | --- |
| Antibody Name | Catalog No. | Supplier |
| AR (Androgen Receptor) | 5153 | Cell Signaling Technology, USA |
| P65 | 8242 | Cell Signaling Technology, USA |
| p-P65 | 3033 | Cell Signaling Technology, USA |
| SLC7A11 | 12691 | Cell Signaling Technology, USA |
| GPX4 | 52455 | Cell Signaling Technology, USA |
| SPP1 | ab125066 | Abcam, UK |
| CD44 | 37259 | Cell Signaling Technology, USA |
| CD206 | ab189524 | Abcam, UK |
| 4-HNE | ab46545 | Abcam, UK |
| KLK3 | Proteintech | 10679-1-AP |
| GAPDH | ab128915 | Abcam, UK |
| β-actin | AC004 | Abclonal, China |
| Lamin B1 | ab133741 | Abcam, UK |
| Ki-67 | sc-23900 | Santa Cruz |
| Anti-rabbit IgG (HRP) | 7074 | CST |
| Anti-mouse IgG (HRP) | 7076 | CST |
| Anti-rabbit IgG (HRP) | ab6721 | Abcam, UK |
| Anti-mouse IgG (HRP) | ab6728 | Abcam, UK |
| HA Tag Antibody | 51064-2-AP | Proteintech |
| His Tag Antibody | 10001-0-AP | Proteintech |
| Anti-FLAG IP/Co-IP Kit | P2181M | Beyotime |

| **Table S2** | | | | | |
| --- | --- | --- | --- | --- | --- |
| **Application** | **Gene** | **Species** | | **Forward (5′–3′)** | **Reverse (5′–3′)** |
| **qPCR primer sequence** | AR | Human | | TCCATCTTGTCGTCTTCGGAA | GGGCTGGTTGTTGTCGTGT |
|  | SLC7A11 | Human | | GCCCAAGGGGAGACACAAAAT | AGGTTCTTTGCCGTGCTAAC |
|  | RELA (P65) | Human | | ATGTGGAGATCATTGAGCAGC | CCTGGTCCTGTGTAGCCATT |
|  | SPP1 | Human | | CTCCATTGACTCGAACGACTC | CAGGTCTGCGAAACTTCTTAGAT |
|  | SPP1 | Mouse | | AGCAAGAAACTCTTCCAAGCAA | GTGAGATTCGTCAGATTCATCCG |
|  | CD44 | Human | | CTGCCGCTTTGCAGGTGTA | CATTGTGGGCAAGGTGCTATT |
|  | GAPDH | Human | | AGAAGGCTGGGGCTCATTTG | AGGGGCCATCCACAGTCTTC |
|  | CD206 | Human | | GCAGAAGGAGTAACCCACCC | TGGCAAATGAAGGCGTTTGG |
|  | CD206 | Mouse | | GGCGAGCATCAAGAGTAAAGA | CATAGGTCAGTCCCAACCAAA |
|  | CD163 | Mouse | | GGCTAGACGAAGTCATCTGCAC | CTTCGTTGGTCAGCCTCAGAGA |
|  | CD163 | Human | | AAAGAAGCAGAGTTTGGTCA | AGGTATCTTAAAGGCTCACTG |
| **shRNA targeting sequence** |  |  | |  |  |
|  | shAR#1 | Human | | CCGGCCTGCTAATCAAGTCACACATCTCGAGA TGTGTGACTTGATTAGCAGGTTTTT |  |
|  | shAR#2 | Human | | CCGGCACCAATGTCAACTCCAGGATCTCGAGAT CCTGGAGTTGACATTGGTGTTTTT |  |
|  | shP65#1 | Human | | UCUUUCUGCACCUUGUCGCtt |  |
|  | shP65#2 | Human | | GCGACAAGGUGCAGAAAGAtt |  |
|  | shSLC7A11#1 | Human | | ATAATAAAGAGATAATACG |  |
|  | shSLC7A11#2 | Human | | ATATATGTGTAATGACCTC |  |
|  | shNC | Human | | CCGGCAACAAGATGAAGAGCACCAACTCGAG TTGGTGCTCTTCATCTTGTTGTTTTT |  |
|  | shRELA#1 | Human | | GCCTTAATAGTAGGGTAAGTT |  |
|  | shRELA#1 | Human | | CGGATTGAGGAGAAACGTAAA |  |
| **ChIP-qPCR primer sequence** |  |  | |  |  |
|  | AR Promoter | Human | | AAGAGTGGAGGGAGGATG | TTGAGAGTAAGTTTCTGC |
|  | SLC7A11 Promoter | Human | | CACCGATGAGCTTGATCGCAAGTTC | AAACGAACTTGCGATCAAGCTCATC |
| **Sequences of the AR promoter core binding sequence used in luciferase reporter assays.** | | | | | |
| **pGL3-AR-WT:** GGATAAAACCCGATGGTCACCATTTTCCAAAGGTCAGCTCATCCTGGCTTTCCAGAGCAAAGAGCTAGGGAAGACTTTATTAATAAATCCCTCTTGAAG | | | **pGL3-AR-Mut:** GGATAAAACCCGATGGTCACCATTTTCCAAAGGTCAGCTCATCAGTTAGGGCCAGAGCAAAGAGCTAGGGAAGACTTTATTAATAAATCCCTCTTGAAG | | |
